# Structure-Based Network Analysis of Activation Mechanisms in the ErbB Family of Receptor Tyrosine Kinases: The Regulatory Spine Residues Are Global Mediators of Structural Stability and Allosteric Interactions

**DOI:** 10.1371/journal.pone.0113488

**Published:** 2014-11-26

**Authors:** Kevin A. James, Gennady M. Verkhivker

**Affiliations:** 1 School of Computational Sciences and Crean School of Health and Life Sciences, Schmid College of Science and Technology, Chapman University, Orange, California, United States of America; 2 Department of Pharmacology, University of California San Diego, La Jolla, California, United States of America; University of Georgia, United States of America

## Abstract

The ErbB protein tyrosine kinases are among the most important cell signaling families and mutation-induced modulation of their activity is associated with diverse functions in biological networks and human disease. We have combined molecular dynamics simulations of the ErbB kinases with the protein structure network modeling to characterize the reorganization of the residue interaction networks during conformational equilibrium changes in the normal and oncogenic forms. Structural stability and network analyses have identified local communities integrated around high centrality sites that correspond to the regulatory spine residues. This analysis has provided a quantitative insight to the mechanism of mutation-induced “superacceptor” activity in oncogenic EGFR dimers. We have found that kinase activation may be determined by allosteric interactions between modules of structurally stable residues that synchronize the dynamics in the nucleotide binding site and the αC-helix with the collective motions of the integrating αF-helix and the substrate binding site. The results of this study have pointed to a central role of the conserved His-Arg-Asp (HRD) motif in the catalytic loop and the Asp-Phe-Gly (DFG) motif as key mediators of structural stability and allosteric communications in the ErbB kinases. We have determined that residues that are indispensable for kinase regulation and catalysis often corresponded to the high centrality nodes within the protein structure network and could be distinguished by their unique network signatures. The optimal communication pathways are also controlled by these nodes and may ensure efficient allosteric signaling in the functional kinase state. Structure-based network analysis has quantified subtle effects of ATP binding on conformational dynamics and stability of the EGFR structures. Consistent with the NMR studies, we have found that nucleotide-induced modulation of the residue interaction networks is not limited to the ATP site, and may enhance allosteric cooperativity with the substrate binding region by increasing communication capabilities of mediating residues.

## Introduction

**Figure 1:**
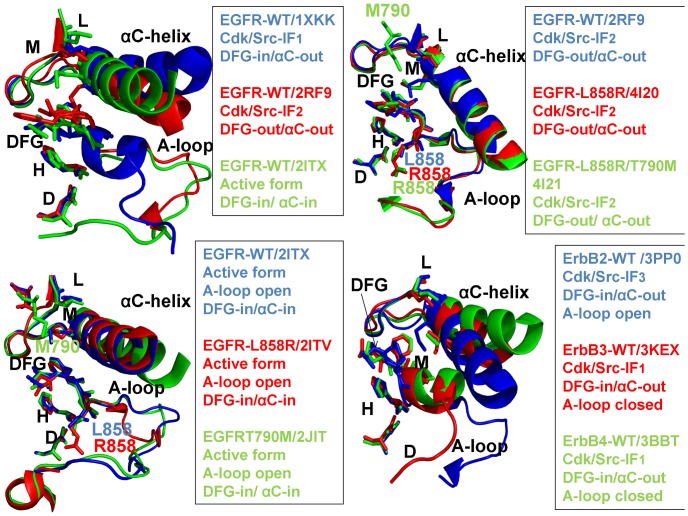
Structural Characteristics of the ErbB Kinases. The crystal structures of the ErbB kinase family in different functional states are depicted using a comparison of key regulatory regions in the catalytic domain. The three regulatory elements of the kinase domain shown are the αC-helix, the DFG-Asp motif (DFG-Asp in, active; DFG-Asp out, inactive), and the activation loop (A-loop open, active; A-loop closed, inactive). In Cdk/Src inactive structures the αC-helix is displaced outwards the N-terminal lobe adopting a αC-out (swung-out) conformation that inhibits the formation of the active enzyme form. The R-spine residues (M766, L777, H835, F856, and D896) and the DFG motif are shown in colored sticks. Note that the R-spine residues in a different sequence numbering of the EGFR kinase domain correspond to M742, L753, H811, F832, and D872 residues. Left Upper Panel. Structural differences in the functional regions of the EGFR-WT crystal structures: Cdk/Src-IF1 state (in blue), DFG-in/αC-helix-out (pdb id 1XKK, 2GS7); Cdk/Src-IF2 conformation (in red), DFG-out/αC-helix-out (pdb id 2RF9); and the active conformation (in green), DFG-in/αC-helix-in (pdb id 2ITX, 2J6M). Right Upper Panel. Structural similarities in the functional regions of the Cdk/Src-IF2 EGFR-WT conformation (in blue), DFG-out/αC-helix-out (pdb id 2RF9); Cdk/Src-IF2 EGFR-L858R conformation (in red), DFG-out/αC-helix-out (pdb id 4I20); and Cdk/Src-IF2 EGFR-L858R/T790M double mutant conformation (in green), DFG-out/αC-helix-out (pdb id 4I21). Left Lower Panel. Structural similarities in the functional regions of the active EGFR-WT conformation (in blue), DFG-in/αC-helix-in (pdb id 2ITX, 2J6M); the active EGFR-L858R conformation (in red), DFG-in/αC-helix-in (pdb id 2ITV); and the active EGFR-T790M conformation (in green), DFG-in/αC-helix-in (pdb id 2JIT). Right Lower Panel. Structural differences in the functional regions of Cdk/Src-IF3 ErbB2-WT conformation (in blue), DFG-in/αC-helix-out, A-loop open (pdb id 3PP0); Cdk/Src-IF1 ErbB3-WT conformation (in red), DFG-in/αC-helix-out, A-loop closed (pdb id 3KEX, 3LMG); and Cdk/Src-IF1 ErbB4-WT conformation (in green), DFG-in/αC-helix-out, A-loop closed (pdb id 3BBT).

The human protein kinases play a fundamental regulatory role in orchestrating functional processes in complex cellular networks [1]–[3]. The mechanisms that regulate catalytic activities of protein kinases include phosphorylation, autoinhibition and allosteric activation by binding partners [4]. The diversity of structural mechanisms that regulate a dynamic switch between inactive and active kinase forms may involve several layers of allosteric control that enable various kinase functions [5]–[16]. The crystal structures of protein kinases in different functional states have underscored the role of specific regions in the catalytic domain whose structural variations can determine regulatory preferences [17], [18]. The main regulatory elements within the kinase catalytic domain include the αC-helix, the DFG-Asp motif (DFG-Asp in, active; DFG-Asp out, inactive), and the activation loop (A-loop open, active; A-loop closed, inactive) (Figure 1, Table 1). Structural coupling of the DFG motif and the regulatory αC-helix has been long recognized as central in controlling a dynamic equilibrium between major functional forms that include an inactive state (DFG-out/αC-helix-in), a Cdk/Src inactive conformation (DFG-in/αC-helix-out) and an active state (DFG-in/αC-helix-in). Protein kinase regulation is also governed by a dynamic coupling of two spatially distributed networks of mostly hydrophobic residues that form a regulatory spine (R-spine) and a catalytic spine (C-spine) [19]–[21]. The analysis of protein kinase crystal structures has identified that the R-spine and the hydrogen bond networks that connect the N-terminal and the C-terminal kinase lobes may be perturbed and often disrupted in the inactive conformations, whereas a cooperative assembly and stabilization of the spine motifs along with the characteristic salt bridges constitute critical features of activation kinase mechanisms [22].

**Table 1:** The Functional Regions of the ErbB Kinases.

Kinase Domain	EGFR	ErbB2	ErbB3	ErbB4
P-loop GSGAFG	719–724	727–732	697–702	700–705
Catalytic K	K745	K753	K723	K726
Catalytic αC-E	E762	E770	H740	E743
αC-helix	751–769	760–775	738–747	733–749
Hinge motif	792–796	800–804	770–774	773–777
Gatekeeper residue	T790	T798	T768	T771
HRD motif	835-HRD-837	843-HRD-845	813-HRN-815	816-818
A-loop DFG motif	855-DFG-857	863-DFG-888	833-DFG-835	836-DFG-838
P+1 loop WMAPE	880–884	888–892	858–862	861–865
R-spine αC-helix	M766	M774	I744	M747
R-spine β4-Strand	L777	L785	L755	L758
R-spine F (DFG)	F856	F864	F834	F837
R-spine H (HRD)	H835	H843	H813	H816
R-spine αF-helix	D896	D904	D874	D877

The residue ranges of functional regions in the ErbB kinases are based on the crystal structures of EGFR (pdb id 2ITX), ErbB2 (pdb id 3PP0), ErbB3 (pdb id 3LMG), and ErbB4 (pdb id 3BCE).

The ErbB protein tyrosine kinases are among the most important cell signaling families and mutation-induced modulation of their activity is associated with diverse functions in biological networks and human disease [23], [24]. A common regulatory signature of the ErbB kinases is based on sharing a Cdk/Src inactive structure with a characteristically low catalytic activity. Crystal structures of the EGFR catalytic domain in the wild-type (WT) [25]–[27] and mutant forms [28]–[30] have detailed characteristic features of Cdk/Src-IF_1_ (DFG-in/αC-helix-out) and active conformations (DFG-in/αC-helix-in) (Figure 1), demonstrating that oncogenic mutants stabilize the active form of EGFR. The crystal structures of the inhibitory complexes between the EGFR kinase domain and a fragment of the cytoplasmic protein MIG6 [31] have unveiled an alternative Cdk/Src inactive form with DFG-out/αC-helix-out (Cdk/Src-IF_2_) (Figure 1), in which the DFG motif is in the inactive DFG-out position, but the interactions constraining the αC-helix in the inactive position are removed, and the A-loop is in a fully extended conformation (A-loop open) as in the active EGFR structures. Another Cdk/Src inactive conformation (Cdk/Src-IF_3_) was detected in the crystal structure of the ErbB2 kinase where the αC-helix and the DFG motif conform to their DFG-in/αC-helix-out positions, but the A-loop adopts an active, open conformation [32] (Figure 1). The ErbB3 kinase has long been considered as inactive, and classified as a pseudokinase, since the key catalytic residues are conspicuously missing in ErbB3. However, recent crystallographic studies have indicated that the catalytically inactive ErbB3 kinase domain can bind ATP and serve as an activator of the EGFR kinase domain [33]. The crystal structure of the catalytically inactive ErbB3 kinase domain has revealed a Cdk/Src-IF_1_ conformation that is similar to that of EGFR and ErbB4 kinases, albeit with a shortened αC-helix [33]. Subsequent studies have reported a crystal structure of the ErbB3 kinase domain bound to an ATP analogue and have demonstrated that human ErbB3 kinase can bind ATP and retain sufficient kinase activity, though ∼1000-fold less than the canonical ErbB kinases [34]. Crystal structures of the ErbB4 kinase domain in the active and inhibited Cdk/Src-IF_1_ forms [35], [36] have suggested that structural determinants of kinase activation may be conserved among the EGFR and ErbB4 catalytic domains. Crystallographic studies of the ErbB kinase domains have also discovered that the formation of an asymmetric dimer between the C-lobe of a “donor” (activator) monomer and the N-lobe of an adjacent “acceptor” (receiver) monomer is a common structural mechanism required to achieve full kinase activation [37]–[40].

A number of human cancers are associated with mutations causing the increased expression of the ErbB kinases. More than 200 activating and drug resistance EGFR mutations have been reported [41], and molecular mechanisms of mutation-induced kinase activation have been extensively discussed [42], [43]. Oncogenic kinase mutants have been long linked with their ability to lock the catalytic domain in a constitutively active state - a functional form whose uncontrollable activity may contribute to the initiation or progression of cancer [44], [45]. Recent crystallographic studies [46], [47] have discovered that the catalytic domains of the EGFR-L858R and EGFR-L858R/T790M mutants in the inactive form can adopt a mobile Cdk/Src-IF_2_ conformation (DFG-out/αC-helix-out) that may facilitate conformational release from the inactive dormant state, resulting in an accumulation of a constitutively active form and elevated enzyme activities. Biochemical reconstitution analysis in combination with the crystal structure of an asymmetric dimer of the L858R/T790M mutant [48], [49] have revealed a new mechanism of mutant-specific kinase regulation in which oncogenic EGFR mutants can preferentially assume the acceptor role in the regulatory dimers.

Structural and computational approaches have been instrumental in revealing the atomic details of protein kinase dynamics at different levels of complexity: from detailed analyses of the catalytic domain to simulations of the regulatory dimer assemblies. A significant body of computational studies has focused on elucidating molecular mechanisms of the ErbB kinases [50]–[59]. Molecular dynamics (MD) simulations and the energy landscape analysis have investigated the structural and energetic basis of mutation-induced changes in the EGFR kinase domain [60], [61]. These studies have determined that the inactive EGFR-WT state is more stable than the active state, and the L858R mutation could differentially perturb both active and inactive conformations to shift thermodynamic preferences towards the activated form. Recently, simulation boundaries have been pushed to new unprecedented levels of multiple microsecond simulations, revealing that the catalytic domain of EGFR may sample a locally disordered state and that oncogenic mutations could reduce the disorder in the αC-helix region of the dimerization interface, thus promoting acquisition of an active asymmetric dimer and stabilization of a constitutively active form [62]. Subsequent multi-scale simulations have witnessed spontaneous conformational transitions between the inactive and active states via locally disordered intermediate conformations, whose functional relevance was independently confirmed by hydrogen exchange mass spectrometry (HX-MS) experiments [63], [64]. The effects of oncogenic mutations on the conformational landscape of the EGFR kinase have been also quantified in another series of large-scale computer simulations [65]. These studies have similarly concluded that mutation-induced alterations in the relative stability of the kinase states and the reduction of disorder at the dimer interface may serve as catalysts of kinase activation by oncogenic mutations. Recent investigations have combined multi-scale molecular simulations with structure-functional approaches to demonstrate that the activation mechanism may involve a cooperative effect of the external, internal, and transmembrane segments of the complex EGFR assembly [66], [67].

The complex changes seen in the energy landscapes of protein kinases obtained from X-ray crystallography, NMR studies, and large-scale computer simulations reflect global changes in the residue interaction networks that can modulate allosteric coupling between regulatory regions. The free energy landscape analysis of kinase mechanisms has emphasized that the relative populations of preexisting conformational states and allosteric communication pathways can be effectively modulated by activation mutations and controlled by a small number of “privileged” functional residues [68]. Understanding how conformational equilibrium between functional kinase states can be altered and redistributed upon ligand binding and/or mutations is critical for quantifying molecular basis of allosteric regulation. Structural studies [69], [70], NMR spectroscopy investigations [71]–[76] and computer simulations [77], [78] of cAMP-dependent protein kinase A (PKA-C) have confirmed the existence of multiple functional forms and allosteric interaction networks that can regulate conformational equilibrium between dynamically committed, uncommitted, and quenched states. ATP binding can redistribute the relative populations of these states and activate a nucleotide-bound functional form of PKA-C that is structurally and dynamically committed to catalysis [71]–[76]. These NMR studies have also discovered the effect of positive allosteric cooperativity in PKA-C, according to which ATP binding in the nucleotide binding site can enhance the substrate affinity in the allosteric site, thus confirming that the interaction networks and long-range communication between distal kinase regions may control catalytic reaction and mediate substrate recognition.

The residue interaction networks can be described as weighted graphs providing a convenient and robust framework for understanding allosteric communications in protein systems [79], [80]. Structure-based network models often employ common measures of node centrality (degree, closeness, and betweenness) to characterize local and global connectivity of residues [81]–[83]. Integration of molecular dynamics simulations and protein structure network analysis has been successfully used to identify functionally important regulatory sites and model allosteric communication pathways for a variety of protein systems [84]–[88]. These studies have shown that the residue interaction networks in protein structures can be characterized by small-world organization, in which local interactions and long-range coupling between mediating nodes are properly balanced to achieve an optimal trade-off between network resilience and efficiency [89]–[91]. These networks are efficient in transmitting long-range signal due short paths between any pair of nodes, but may become vulnerable to targeted attacks on a small number of central nodes.

In this work, atomistic simulations were combined with the ensemble-based network analysis to characterize evolution of the residue interaction networks in the ErbB kinases during conformational equilibrium changes. Conformational dynamics of the ErbB kinases was analyzed in different functional states by simulating multiple crystal structures of the catalytic domain and regulatory dimer complexes. We also investigated the allosteric effect of ATP binding on conformational dynamics and structural stability of the EGFR structures. This study shows that structural stability and allosteric interactions in the ErbB kinase family can be mediated by a small number of sparsely distributed high centrality nodes that correspond to the conserved functional residues in the R-spine, the regulatory HRD and DFG motifs, and the substrate binding P+1 loop. We demonstrate that the optimal communication pathways may be controlled by these nodes and ensure efficient long-range signaling in the functional kinase states. This study reveals that the residue interaction networks in the kinase structures may exhibit elements of modularity that may have evolved to achieve a trade-off between structural stability, the efficiency of allosteric communications and resilience against perturbations in the protein environment.

## Results

### Conformational Dynamics of the ErbB Kinases: Structural Stability of Regulatory Regions

**Figure 2:**
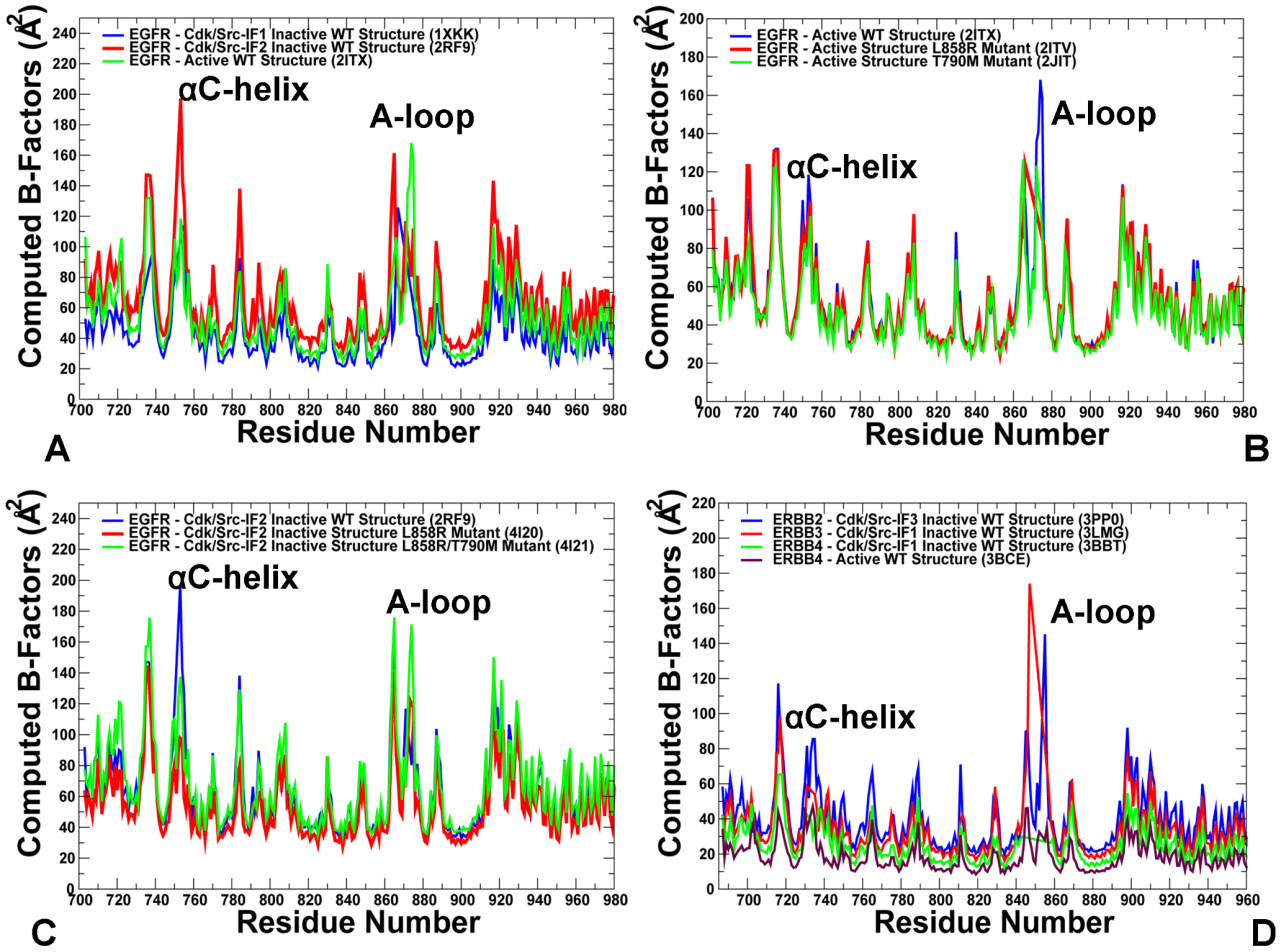
Residue-Based Equilibrium Fluctuations of the ErbB Kinases. (A) The computed B-factors describe time-averaged residue fluctuations obtained from simulations of Cdk/Src-IF1 EGFR-WT (pdb id 1XKK, in blue), Cdk/Src-IF2 EGFR-WT structure (pdb id 2RF9, in red), and the active EGFR-WT structure (pdb id 2ITX, in green). (B) The computed B-factors obtained from simulations of the active structures of EGFR-WT (pdb id 2ITX, in blue), EGFR-L858R (pdb id 2ITV, in red), and EGFR-T790M (pdb id 2JIT, in green). C) The computed B-factors for Cdk/Src-IF2 structures of EGFR-WT (pdb id 2RF9, in blue), EGFR-L858R (pdb id 4I20, in red), and EGFR-L858R/T790M (pdb id 4I21, in green). (D) The computed B-factors obtained from simulations of Cdk/Src-IF3 ErbB2-WT structure (pdb id 3PP0, in blue), Cdk/Src-IF1 ErbB3-WT structure (pdb id 3LMG, in red), Cdk/Src-IF1 ErbB4-WT structure (pdb id 3BBT, in green), and the active ErbB4-WT structure (pdb id 3BCE, in maroon).

MD simulations of the ErbB kinases were performed using multiple crystal structures of the catalytic domain and regulatory dimer complexes in the normal and oncogenic forms. We analyzed equilibrium simulations of the EGFR structures and asserted that conformational dynamics of the regulatory regions is conserved in the active kinase forms, but may vary significantly depending on the inactive kinase state. Equilibrium fluctuations in the inactive and active EGFR structures displayed characteristic differences in the conformational mobility and coordinated motions of the P-loop, αC-helix, and the A-loop (Figure 2A). These regions displayed larger thermal fluctuations and the increased conformational flexibility in the active kinase form as evident from the root mean square fluctuation (RMSF) of the backbone residues and computed B-factors. However, the inactive Cdk/Src-IF_1_ form showed smaller thermal variations in the key functional regions, mainly due to the autoinhibitory interactions between the P-loop, a helical motif within the A-loop, and the αC-helix. The greater structural rigidity of the Cdk/Src-IF_1_ structure may lock EGFR in the autoinhibited dormant state, thereby decreasing the probability of inadvertent activation. In contrast, a markedly greater flexibility could be seen in the Cdk/Src-IF_2_ structure, where thermal motions were especially pronounced in the αC-helix, the αC-β4-loop, and the A-loop (Figure 2A). In this structure, the autoinhibitory constraints are removed, leading to the increased flexibility and decoupling of the αC-helix and the A-loop movements. A more uniform pattern of small thermal fluctuations was detected across all regions in the active EGFR structures (Figure 2B). The atom-based fluctuation profiles of the EGFR structures depicted a more detailed view of variations experienced by the P-loop, αC-helix, and the A-loop regions (Figure S1). These equilibrium profiles similarly highlighted the increased flexibility of the inactive Cdk/Src-IF_2_ form of EGFR as compared to the autoinhibited and active EGFR structures (Figure S1). Simulations of the EGFR-L858R and the EGFR-L858R/T790M mutants in the inactive Cdk/Src-IF_2_ structure revealed not only local adjustments near the mutational sites, but also long-range changes in the αC-helix and the N-terminal lobe (Figure 2C). In this case, we also observed additional intermediate conformations that were similar to the Cdk/Src-IF_2_ form (DFG-out/αC-helix-out, A-loop open), but in which the αC-helix drifted away from the inactive conformation towards an active αC-helix-in position. At the same time, the effect of L858R and L858R/T790M mutations on conformational dynamics of the active EGFR structure was stabilizing and relatively moderate (Figure 2B). As a result, oncogenic EGFR mutants may escape from the autoinhibitory trap and induce the increased mobility of the inactive conformations by populating a rapidly interconverting region of the conformational landscape. This could facilitate fast conformational transitions between the inactive Cdk/Src-IF_2_ state and the active EGFR form, ultimately leading to stimulated activities of oncogenic EGFR mutants. MD simulations of the inactive ErbB2 crystal structure (Cdk/Src-IF_3_ conformation with DFG-in/αC-helix-out, A-loop open) (Figure 2D) revealed a significant conformational mobility in the P-loop, αC-helix and A-loop regions. Unlike other members of the ErbB family, the increased conformational flexibility in the ErbB2 structure was spread beyond the αC-helix region suggesting a partial weakening of the entire catalytic core.

The collective motions of the ErbB kinase structures were evaluated using principal component analysis (PCA) [92], [93]. According to a comprehensive account of PCA applications in protein dynamics, MD simulations at the nanosecond time scale may be sufficient for an effective separation of time scales and an adequate characterization of essential dynamics [94]. This study has also noted that PCA of protein conformational dynamics based on the heavy atoms, as opposed to C_α_ atoms only, can provide a better description of slow degrees of freedom and yield a more accurate view of global collective motions. Consistent with these arguments, we utilized the extended set of backbone heavy atoms (N, C_α_, C_β_, C, O) in the PCA modeling of the ErbB kinase structures. We observed that almost the same shapes can be obtained for the slowest modes of motion when using between 3 and 10 lowest frequency modes. Based on our computations, the 10 lowest eigenvectors captured between 85% and 90% of the total variance in the collective kinase motions, while the essential subspace covered by the first three lowest PCA modes typically accounted for at least 75% of atomic fluctuations in each trajectory and approached 80% in simulations of the active kinase structures. These results are in line with earlier findings [94] indicating that the heavy atom-based PCA may provide a sufficient convergence of high frequency subspaces and thus allow for a better separation of orthogonal low frequency modes. Indeed, we found that a relatively small number of low frequency modes may describe most of the slow conformational motions. Based on these results and to streamline functional dynamics analysis, we characterized conformational dynamics and collective movements of the ErbB kinases in the essential space of the three low frequency modes. For all catalytic domain structures, the first principal mode typically corresponded to the opening and closing movements of the N-terminal and C-terminal lobes with respect to each other. In the second principal mode, the kinase lobes displayed a shear motion between the N-terminal and C-terminal lobes, in which a sliding movement of the lobe interface corresponded to the forward displacement of one lobe and inward displacement of the other lobe. In another principal mode, the C-terminal and N-terminal tails rotated in opposite directions with respect to the C-terminal lobe. The observed pattern of principal motions is conserved among protein kinase folds [95] and consistent with the NMR studies of conformational dynamics in protein kinases [71]–[77].

**Figure 3:**
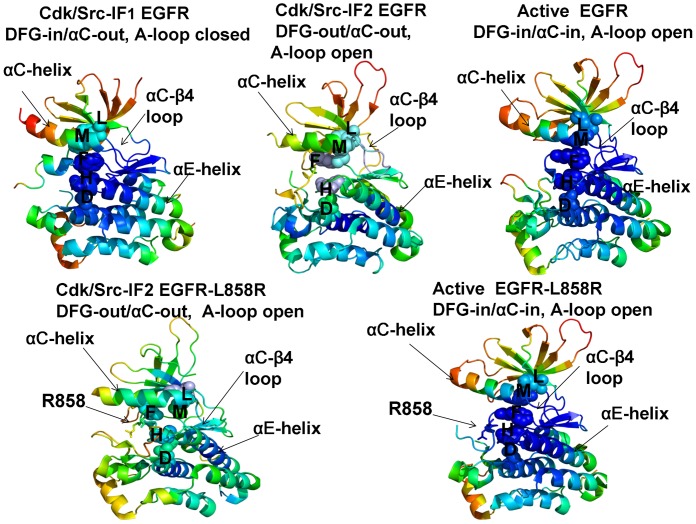
Conformational Mobility Analysis of the EGFR-WT and EGFR-L858R Kinases. Conformational mobility profiles of EGFR-WT are shown for the inactive Cdk/Src-IF1 form (pdb id 1XKK, left upper panel), the inactive Cdk/Src-IF2 state (pdb id 2RF9, middle upper panel) and the active conformation (pdb id 2ITX, right upper panel). Conformational mobility of EGFR-L858R is shown for the Cdk/Src-IF2 form (left lower panel) and the active conformation (right lower panel). The backbone heavy atoms (N,C_α_,C_β_,C,O) were employed for the PCA computations. Conformational dynamics profiles were computed by averaging protein motions in the space of three lowest frequency modes. The color gradient from blue to red indicates the decreasing structural rigidity (or increasing conformational mobility) of the protein residues and refers to an average value over the backbone atoms in each residue. The functional kinase regions αC-helix, αC-β4-loop, and αE-helix as well as the R-spine residues are annotated and their positions are indicated by arrows. The R-spine residues are also highlighted in spheres and colored according to their degree of structural stability. Conformational mobility profiles were obtained from simulations of complete structures, where unresolved segments and disordered loops were modeled with the ModLoop server [127], [128]. These profiles were mapped onto the original crystal structures of EGFR for clarity of presentation.

**Figure 4:**
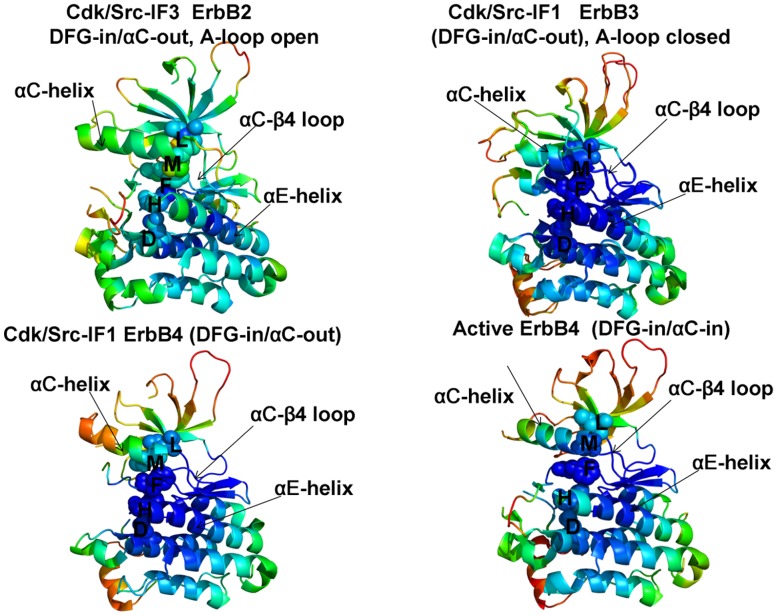
Conformational Mobility Analysis of the ErbB Kinases. Conformational mobility mapping of ErbB2-WT in the inactive Cdk/Src-IF3 form (left upper panel), ErbB3-WT in the inactive Cdk/Src-IF1 conformation (right upper panel), ErbB4-WT in the Cdk/Src-IF1 form (left lower panel) and the active form (right lower panel). The backbone heavy atoms (N, C_α_, C_β_, C, O) were employed for the PCA calculations. Conformational dynamics profiles were computed by averaging protein motions in the space of three lowest frequency modes. The color gradient from blue to red indicates the decreasing structural rigidity (or increasing conformational mobility) of the protein residues and refers to an average value over the backbone atoms in each residue. The key functional regions αC-helix, αC-β4-loop, and αE-helix as well as the R-spine residues are annotated and their positions are indicated by arrows as in Figure 3. The conformational mobility profiles were mapped onto the original crystal structures of ErbB kinases.

**Figure 5:**
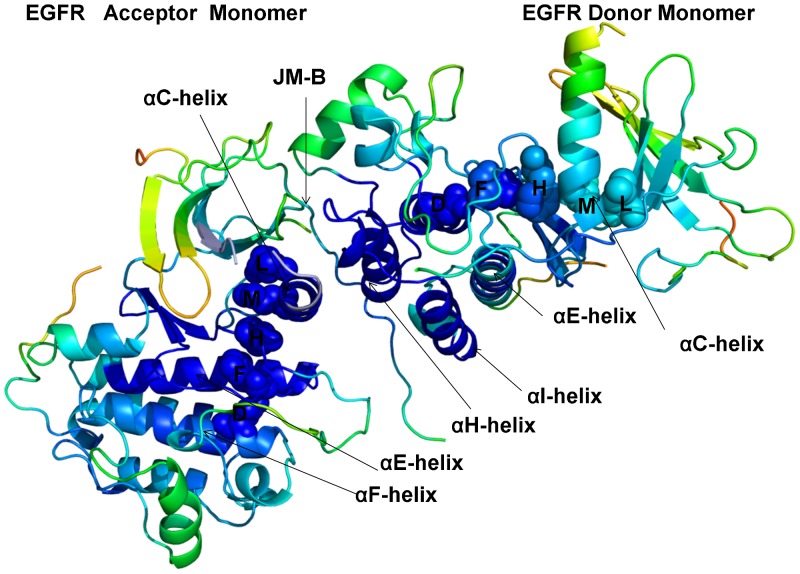
Conformational Mobility Profile of the Active EGFR Dimer. Structural distribution of conformational mobility in the asymmetric active dimer of EGFR-WT. In an asymmetric dimer arrangement a donor monomer interacts with an acceptor through interactions involving the αH-helix and αI-helix of the donor as well as the JM-B segment and the αC-helix of the acceptor. The key functional regions are annotated and pointed to by arrows as in Figures 3, 4. Note the increased stability of the acceptor monomer, particularly a uniform stabilization of the R-spine residues in the acceptor subunit. The conformational mobility profiles were mapped onto the original crystal structure of the active EGFR dimer.

Conformational dynamics profiles were mapped onto the ErbB structures where the residue mobility referred to an average B-factor value computed over backbone atoms in each given residue (Figures 3, 4). The distributions revealed that structural rigidity of the αC-β4 loop can be linked to the positional variability of the αC-helix. The “boundary” between the rigid αC-β4 loop and a more flexible αC-helix can define a functional hinge connecting regions of high and low structural stabilities. This dynamic signature is conserved among functional kinase states and may be exploited to promote global conformational changes between the inactive and active structures. Conformational mobility map of the ErbB2 structure (Figure 4) demonstrated the increased conformational mobility in all regions of the catalytic domain. Noticeably, structural stability of the αC-β4-loop, αC-helix, and the R-spine residues was compromised in the inactive ErbB2 structure. The more restricted thermal fluctuations in the inactive ErbB3 kinase were reminiscent of those in the autoinhibitory form of EGFR. Despite a shortened αC-helix in the crystal structures of ErbB3, the catalytic core and the αC-β4/αC-helix region were rigid. The obtained dynamic profile of the ErbB3 kinase corroborates with structural studies [33], [34] that attributed the lack of the ErbB3 catalytic activity to its overly stable inactive form. To characterize patterns of structurally stable and flexible regions in the functional kinase forms, we analyzed conformational dynamics of the R-spine residues. The EGFR R-spine includes L777 from the β4-strand, M766 from the C-terminal end of the αC-helix, F856 of the DFG motif in the activation segment, H835 of the HRD motif of the catalytic loop, and D896 of the αF-helix (Table 1). The backbone of H835 is anchored to the αF-helix via hydrogen bonding to D896. The substrate binding P +1 loop, the A-loop, and the αH-αI loop bind to the αF-helix forming a dense interaction network. While the R-spine residues of active EGFR are linearly connected, the αC-helix position in the inactive EGFR confirmation leads to a dislocation between M766 and L777 residues and partly disassembled spine architecture. A common dynamics signature of the active structures was a uniform structural stability acquired by all spine residues that integrate coordinated movements of the αC-β4-loop, αC-helix, αE-helix, and αF-helix in their active positions. A partially assembled architecture of the hydrophobic spine in the autoinhibitory structures of EGFR, Erbb3 and ErbB4 is fairly constrained due to structural rigidity of the spine residues, thus increasing the energetic cost of inducing the active conformation. In contrast, the regulatory regions are fairly dynamic and the R-spine structure is loose in the mobile Cdk/Src-IF2 conformations that are adopted by the EGFR mutants. Among interesting findings of this analysis was a striking similarity between functional dynamics profiles of the catalytic domains of EGFR (Figure 3) and ErbB4 (Figure 4). The R-spine residues in EGFR (M766, L777, H835, F856, D896) and ErbB4 (M747, L758, H816, F837, D877) have a very similar profile, revealing structural stability of the HRD and DFG motifs, whereas the αC-β4/αC-helix interface residues (M766, L777 in EGFR and M747, L758 in ErbB4) mark the border between regions of high and low structural stability. The dynamics profile of the EGFR dimer (Figure 5) revealed the increased stability of the acceptor monomer that extended beyond the interface, suggesting that the formation of an asymmetric complex may allosterically strengthen structural integrity of the active kinase form. The R-spine residues in the acceptor monomer become structurally stable and effectively immobilized in their active positions. This is consistent with the notion that EGFR and ErbB4 kinases employ the same autoinhibitory mechanisms [39], [40]. Hence, conformational dynamics of the ErbB kinases underscored structural stability of the inactive Cdk/Src-IF_1_ structure (DFG-in/αC-helix-out, A-loop closed) that could be contrasted with the conformationally mobile Cdk/Src-IF_2_ state (DFG-out/αC-helix-out, A-loop open) and Cdk/Src-IF_3_ conformations (DFG-in/αC-helix-out, A-loop open).

### Structural Stability Profiles of the Kinase Catalytic Doman and Active Regulatory Dimer: The Force Constant Analysis

**Figure 6:**
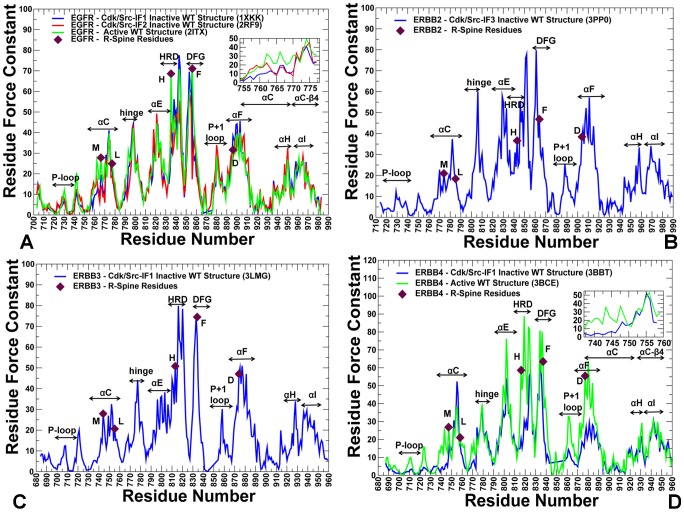
The Force Constant Profiles of the Kinase Catalytic Domain. Dynamics-based analysis of structural stability in the ErbB crystal structures. (A) The residue-based force constant profiles of the EGFR-WT crystal structures: Cdk/Src-IF1 conformation (in blue), Cdk/Src-IF2 conformation (in red), and the active conformation (in green). A close-up view of the EGFR force constant profile in the αC-helix (residues 752–768) and the adjacent αC-β4-loop regions (residues 769–777) is provided as an inset. (B) The force constant profile of Cdk/Src-IF3 ErbB2 structure. (C) The force constant profile of Cdk/Src-IF1 ErbB3 structure. (D) The force constant profiles of Cdk/Src-IF1 ErbB4 conformation (in blue) and the active ErbB4 conformation (in green). A close-up view of the ErbB4 force constant profile in the αC-helix (residues 735–749) and the adjacent αC-β4-loop (residues 750–758) is provided as an inset. The annotated functional regions included P-loop, αC-helix, hinge, αE-helix, HRD motif, DFG motif, substrate binding P+1 loop, αF-helix, αH, and αI helix. The R-spine residues are indicated by filled maroon-colored diamond symbols. Note that the R-spine residues corresponded to the peaks in the distributions.

In the previous section we asserted that conformational dynamics and functional motions of the ErbB kinases may be associated with allosteric interactions between regulatory regions. Here, we analyzed structural stability of the regulatory regions in different functional states of the ErbB kinases and characterized mutation-induced changes in stability profiles that may be relevant for activation mechanisms. For this analysis, we employed a number of complementary approaches, including the force constant profiling of residue connectivity, the contact network analysis of residue closeness, the relative solvent accessibility (RSA) evaluation of local residue environment, and the network-based analysis of local contact density. In the ensemble-based force constant analysis, the equilibrium fluctuations of the mean distance between each residue and the rest of the protein were converted into force constants that measure the energy cost of the residue displacement during equilibrium simulations [96], [97]. The high force constants are typically associated with structurally stable residues that display small fluctuations in their distances to other residues and often correspond to highly connect and effectively communicating rigid sites. Previous studies have linked structural stability of functionally important residues with their high connectivity, particularly indicating that catalytic and binding site residues typically have high force constant values, which reflects functional constraints imposed on their movement [98], [99]. Abrupt changes between maxima and minima in the force constant profiles may be associated with the regions bridging structurally rigid and flexible regions, often pointing to the hinge sites. The hypothesis tested in our analysis is that the R-spine residues could effectively mediate structural stability and allosteric interactions via regulatory regions. The analysis revealed that high force constant residues in the catalytic domain are assembled near the αC-helix, αE-helix and αF-helix regions (Figure 6), suggesting that structural stability of these structural elements may be critical for allosteric coupling between regulatory regions. In addition, in all functional states, we detected a clear maximum corresponding to the conserved regulatory motif WMAPE (substrate binding P+1 loop) that is anchored to the αF-helix. We also observed abrupt changes in the force constant profiles of the EGFR catalytic domain (Figure 6A) that corresponded to the border between the more flexible αC-helix (residues 751–769) and the more rigid αC-β4 loop (residues 770–777). The force constant values and structural stability of the αC-helix residues were considerably higher in the active EGFR form than in the inactive EGFR conformation. Similar changes were also detected in the force constant profile of the ErbB4 kinase domain (Figure 6D) in which the αC-helix region (residues 733–749) displayed appreciable differences between the inactive and active ErbB4 conformations. At the same time, the force constant values for the αC-β4 loop (EGFR residues 770–777 in Figure 6A and ErbB4 residues 750–758 in Figure 6D) were similar in the inactive and active kinase forms. Accordingly, the hinge site located at the border between the αC-β4 loop (higher structural stability region) and the αC-helix (lower structural stability region) may be involved in modulating large conformational transitions between the inactive and active kinase states. These findings corroborate with the experimental studies in which the αC-β4 loop/αC-helix region was recognized as an intramolecular switch of the ErbB kinase activity [100]. Notably, the αC-β4 loop and the R-spine residues in the inactive form of ErbB2-WT displayed lower force constant values and did not correspond to the distribution peaks (Figure 6B). As a result, structural stability of the regulatory regions a may be compromised in the inactive ErbB2 structure. These factors may contribute to the experimentally observed low catalytic activity of ErbB2 [32]. Hence, the force constant profiles highlighted the conserved features and differences in structural stability of the inactive and active kinase forms.

**Figure 7:**
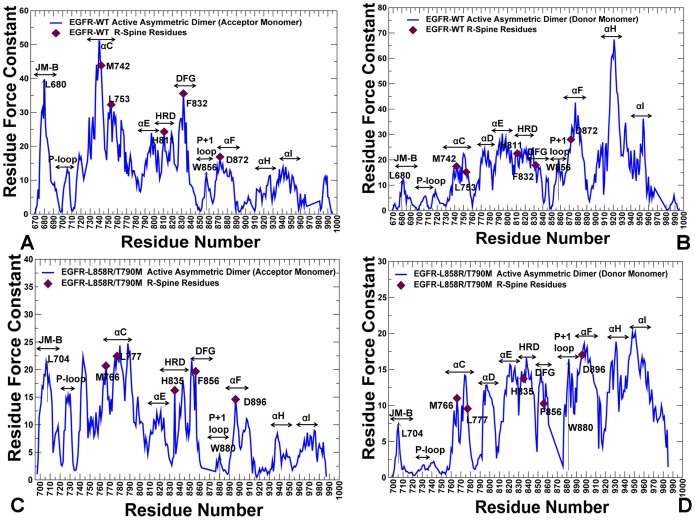
The Force Constant Profiles of the Active EGFR Dimers. Dynamics-based analysis of structural stability in the active asymmetric dimers of EGFR-WT (A, B) and EGFR-L858R/T790M double mutant (C, D). Note that a different EGFR sequence numbering was adopted in these crystal structures and we adhered to the original numbering to streamline the discussion and comparison with the experimental data. The force constant profiles are shown separately for the acceptor monomer (A, C) and donor monomers (B, D). The annotated functional regions included JM-B region, P-loop, αC-helix, hinge, αE-helix, HRD motif, DFG motif, substrate binding P+1 loop, αF-helix, αH, and αI helix. The annotated peaks in the profiles reflecting structural stability of the EGFR-WT dimer included L680 (JM-B region), M742, L753, H811, F832, D872 (R-spine residues), and W856 (P+1 substrate loop). The respective peaks in the profile of the EGFR-L858R/T790M dimer corresponded to L704 (JM-B region), M766, L777, H835, F856, and D896 (R-spine residues), and W880 (P+1 substrate loop). The R-spine residues are indicated by filled maroon-colored diamond symbols. The position of JM-B peaks (L680 in EGFR-WT, L704 in EGFR- L858R/T790M) and P+1 loop peaks (W856 in EGFR-WT, W880 in EGFR- L858R/T790M) are indicated by arrows.

Structural stability of the regulatory residues tends to become considerably more pronounced in the force constant profiles of the EGFR dimer (Figure 7). The noticeable peaks in the acceptor monomer (Figure 7A) corresponded to L680 from the juxtamembrane segment (JM-B segment includes residues 664 to 682) and prominently included the R-spine residues M742 (αC-helix), H811 (HRD motif), F832 (DFG motif), and D872 (αE-helix). Strikingly, the R-spine residues coincided precisely with the highest peaks in the force constant profile of the acceptor monomer, suggesting that these functional residues may serve as global mediators of structural stability in the active EGFR dimer. The stability profile of the donor monomer revealed the important contribution of the αF-helix and the αH-helix, owing to a stable dimer interface that rigidified the position of the αH-helix (Figure 7B). The crystal structure of the L858R/T790M dimer is essentially identical to the EGFR-WT, and structural stability profiles of the acceptor (Figure 7C) and donor monomers (Figure 7D) in the mutant were similar to the respective distributions in EGFR-WT. However, we noticed the emergence of wider peaks in the mutant form of the EGFR dimer. In particular, a broader peak was seen in the αC-helix of the acceptor molecule (Figure 7C), while in the donor molecule the individual peaks corresponding to the αE-helix, HRD and DFG motifs tend to aggregate into a broader maximum (Figure 7D). Similarly, single peaks corresponding to W880 (P+1 substrate site) and D896 (αF-helix) seem to consolidate into a wider maximum (Figure 7D). In our interpretation, this may reflect the integration of structurally stable residues into consolidated modules, pointing to the reorganization of the interaction network and the enhanced structural stability of the mutant dimer. These findings corroborate with the biochemical experiments [48], [49] and provide a useful insight to the mechanism of mutation-induced “superacceptor” activity, which may result from the lower energetic cost of inducing the active conformation in the EGFR mutant relative to EGFR-WT. Because of functional dependency for dimerization it is possible that only the acceptor subunit should be catalytically fully active.

### Probing Residue Environment in the Regulatory Kinase Regions: Local and Global Network Analysis of Residue Connectivity

**Figure 8:**
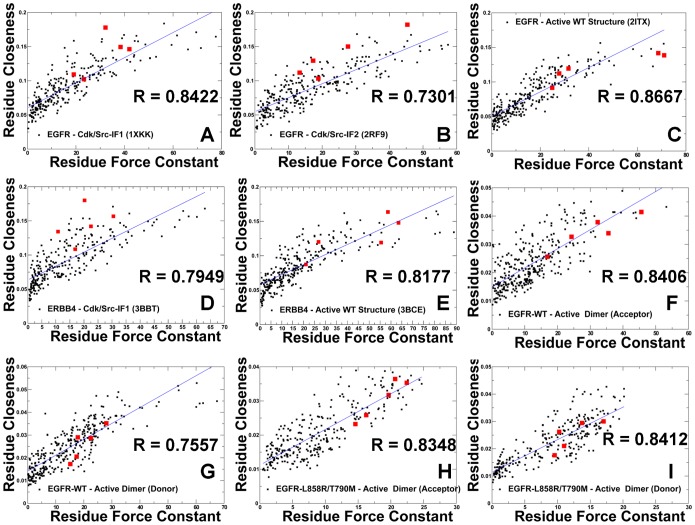
A Comparative Analysis of Residue Connectivity Parameters in the Functional States of the Kinase Domains and Active EGFR Dimers. The scatter graphs between the force constant (a dynamics-based residue connectivity measure) and residue closeness (a network-based residue connectivity measure) values are shown for Cdk/Src-IF1 EGFR-WT (A), Cdk/Src-IF2 EGFR-WT (B), active EGFR-WT (C), Cdk/Src-IF1 ErbB4-WT (D), active ErbB4-WT (E), acceptor monomer of the EGFR-WT dimer (F), donor monomer of the EGFR-WT dimer (G), acceptor monomer of the EGFR-L858R/T790M dimer (H), and donor monomer of the EGFR-L858R/T790M dimer (I). The positions of the R-spine residues are indicated by filled squares colored in red.

We conducted a dynamics-based network analysis in which we utilized the results of MD simulations to determine the distribution of highly connected residues in the ErbB kinases. We employed various network parameters, including the degree of residue node and residue closeness to characterize residue connectivity profiles in the kinase structures. The degree of a residue node is the number of immediate local neighbors in the protein structure and represents a local measure of residue connectivity. The residue closeness corresponds to the inverse of the average of the shortest path between a given residue and all other residues in the protein network and represents a global measure of residue connectivity [81]–[83]. The residues with high closeness can interact directly or indirectly with all other residues of the protein. The degree of a node and closeness are radial measures of network centrality that tend to be highly correlated with each other because they are both based on direct connections. According to our conjecture, high connectivity residues, determined by the consensus of local and global metrics, may correspond to structurally stable sites that are important for kinase function. We first analyzed the relationship between global residue connectivity measures that are represented by the force constant and residue closeness. These parameters are derived from the mean distance of a residue node to all other nodes and thus integrate the effect of the entire protein on a given single residue. By correlating these parameters in different kinase states, we tested whether the R-spine residues could correspond to similar high peaks in these distributions. We found a significant correlation (R∼0.85) between the force constant and the residue closeness values for both the inactive (Figure 8A, B) and active EGFR-WT structures (Figure 8C). A similar level of correlation was also evident in the analysis of the inactive and active forms of ErbB4 (Figure 8D, E). Noteworthy, these global connectivity parameters revealed a significant correlation and cooperativity of the R-spine residues in the active kinase forms, while this relationship was weaker in the inactive structures. In the inactive EGFR form, the αC-helix spine residues (M766, L777) were more flexible, while the HRD and DFG motifs (H835, F856, D896) remained structurally stable. In the active EGFR conformation, we observed the synchronously increased force constant and residue closeness values for the αC-helix and all R-spine residues. This analysis underscored that a uniform structural stabilization of all spine residues could be achieved only in the active dimer (Figure 8 F, G). In this case, both the force constant and residue closeness values of the R-spine residues were generally higher as compared to the other core residues.

We complemented the global analysis of residue connectivity by probing local residue environment using an energetics-based evaluation of relative solvent accessibility (RSA). This approach is rooted in thermodynamic principles of protein stability and demonstrates a strong correspondence with computational and experimental measures of conformational flexibility [101]–[103]. The global RSA values can be used as a simple proxy for predicting intrinsic flexibility and stability of monomeric proteins and the extent of conformational changes that would occur upon complex formation or disassembly [101], [102]. A residue-specific local RSA measure employed here is defined as the ratio of the observed solvent-accessible surface area for a residue to the expected unfolded state value for that amino acid type [104]. According to this model, residues are considered to be solvent exposed if the ratio value exceeds 50% and to be buried if the ratio is less than 20%. As expected, we found that residues with low force constant values are mainly highly exposed (RSA ≥50%), while unexposed amino acids (core, RSA∼0–10%) have high force constant values (Figure S2). It is evident that the solvent-exposed residues have on average lower force constant values and are more flexible than the buried residues. Notably, the force constant and RSA values are only partially related, with a moderate correlation for the EGFR structures (R∼0.5). As a result, these measures of residue connectivity may describe complementary features related to structural stability. Nonetheless, the R-spine residues in the EGFR structures were characterized by both low RSA values (RSA<10%) and high force constants values (Figure S2). The correlation between the force constants and RSA values improved in the active EGFR form (R∼0.55) where the R-spine residues became completely buried (RSA<3%) and attained high force constant values. The divergence in the distributions was apparent in the Cdk/Src-IF2 structures (Figure S2), reflecting the reduced local contact density and the increased solvent exposure of functional residues in this flexible inactive form. In the original hypothesis, we argued that structurally stable regulatory residues could be distinguished by the consensus of both local and global measures of high residue connectivity. To further test this proposal, we determined the local contact density defined by the residue degree in the protein structure network. In particular, we evaluated the distribution of local contacts in the EGFR and ErbB4 structures by specifically focusing on “well-connected” residues with the number of directly interacting neighbors exceeding the selected threshold of four. This analysis indicated that the R-spine residues had on average high local connectivity in both inactive and active EGFR structures (Figure S3). In the inactive conformations, the αC-helix residues (L777 in EGFR, M747 and L758 in ErbB4) had fewer local neighbors and appeared to be more flexible as compared to the rest of the spine. However, all spine residues displayed higher local connectivity and greater stability in the active kinase conformations. The local contact density was generally higher in the regulatory dimers (Figure S4), especially in the αC-helix, αE-helix, and αF-helix of the acceptor monomer. The interaction network of the active dimers is strongly influenced by the JM-B segment and could lead to a denser network near the mediating αC-helix. A number of highly connected residues in the donor monomer corresponded to the αH-helix residues involved in the intermonomer interface (Figure S4). These results indicated that high connectivity of the regulatory residues in the active structures could be amplified by the presence of well-connected neighboring residues with a similar local contact density. The interactions between high connectivity residues with similar node degree are often referred to as the “rich-club” phenomenon [105] which is recognized as a signal of network robustness against random perturbations and mutations. To this end, we explored various structural and energetic measures of residue connectivity in the kinase structures. The global and local measures produced a certain consensus by identifying the R-spine residues as important functional sites that could mediate structural stability of the regulatory regions in the ErbB kinases.

### The Interaction Communities Differentiate between Inactive and Active Forms of the ErbB Kinases

**Figure 9:**
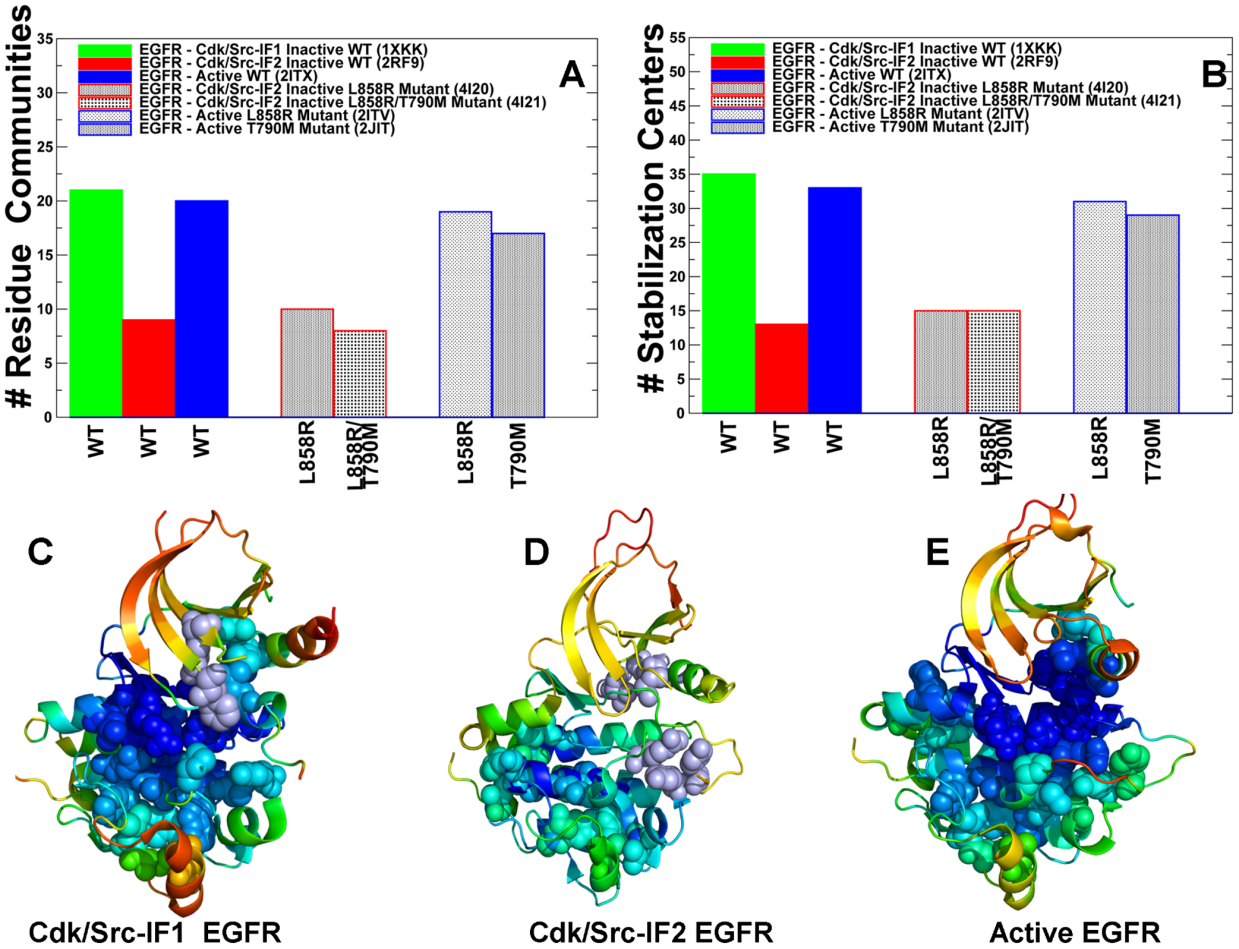
Community Analysis of the EGFR Kinase. The distribution of residue interaction communities (A) and stabilization centers (B) in different functional states of the EGFR kinase. The analysis is based on structurally stable residue interaction networks that were maintained in more than 75% of the simulation samples. The principal interaction communities were mapped onto conformational dynamics profiles of Cdk/Src-IF1 EGFR conformation (C), Cdk/Src-IF2 EGFR conformation (D) and the active EGFR conformation. The communities that are characteristic of different functional states are highlighted in spheres and colored according to structural stability of protein residues. A larger number of stable communities were observed in Cdk/Src-IF1 (C) and active EGFR forms (E).

**Figure 10:**
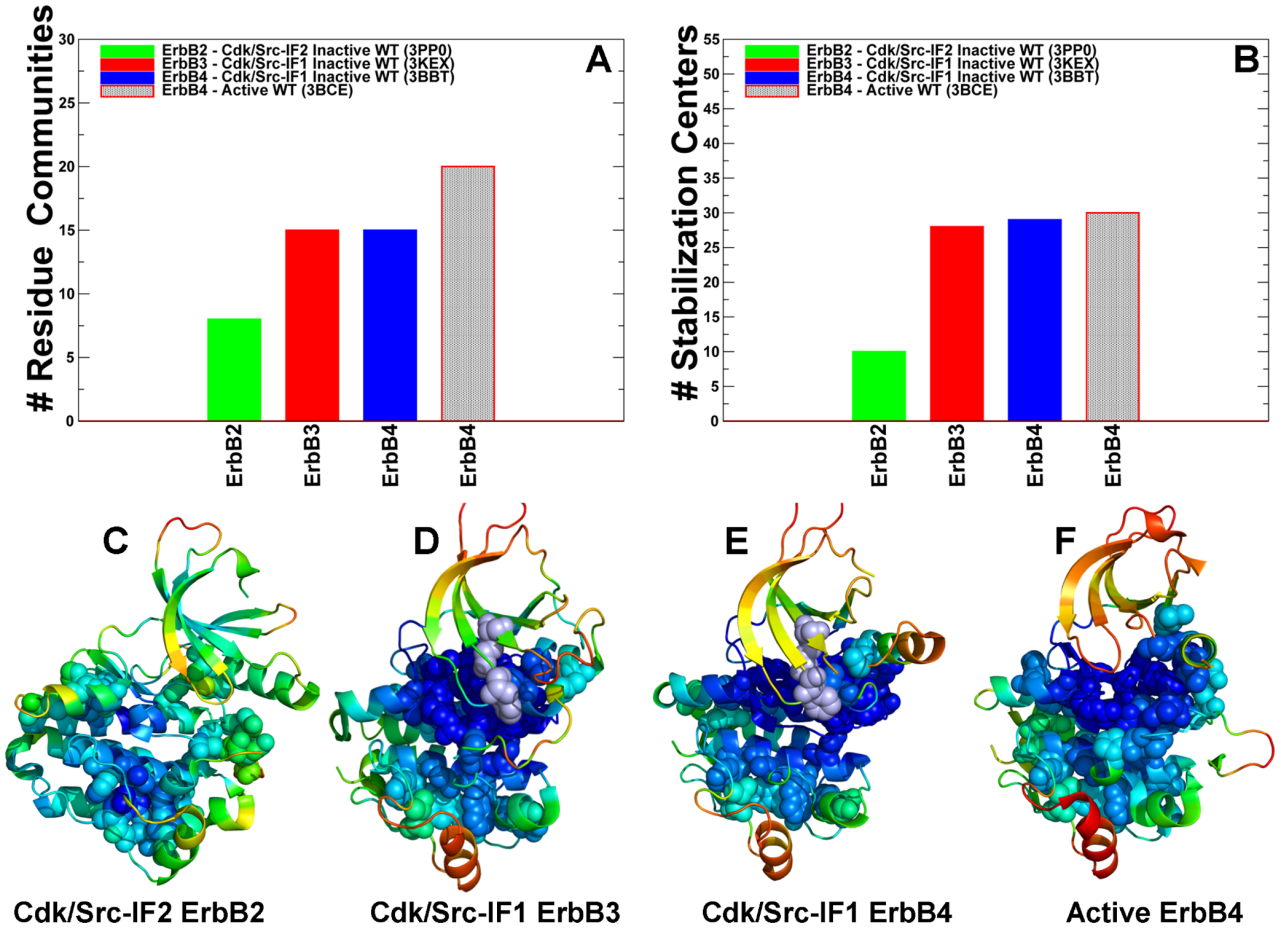
Community Analysis of the ErbB Kinases. The distribution of residue interaction communities (A) and stabilization centers (B) is shown for different functional states of ErbB2, ErbB3, and ErbB4 kinases. The principal interaction communities were mapped onto conformational dynamics profiles of the inactive Cdk/Src-IF3 ErbB2 (C), inactive Cdk/Src-IF1 ErbB3 (D), inactive Cdk/Srdc-IF1 ErbB4 (E), and active ErbB4 (F). The communities that are characteristic of different functional states are highlighted in spheres and colored according to structural stability of protein residues. A larger number of stable communities were observed in the functional states of ErbB4.

Using protein structure network analysis we also characterized the evolution of the residue interaction networks during conformational equilibrium changes in the ErbB kinases. The distribution and the aggregate number of stable interaction communities (Figure 9A) and stabilization centers (Figure 9B) were computed for different functional forms of EGFR. We observed that the number of communities in the inactive EGFR state (Cdk/Src-IF_1_) is greater than in the alternative Cdk/Src-IF_2_ conformation and in the active EGFR-WT state. The interaction communities in the inactive Cdk/Src-IF_1_ state (Figure 9C) have well-defined boundaries and consist of a significant number of residues. Accordingly, strong residue interactions formed within partly overlapping local communities may contribute to the intrinsic rigidity of the autoinhibited EGFR structure and reduce the probability of inadvertent kinase activation. The lower number of small disjointed communities was encountered in the inactive Cdk/Src-IF_2_ conformation, which is consistent with the intrinsic flexibility of this EGFR form (Figure 9D). We observed similarities in the number of communities and stabilization centers for the inactive and active states of EGFR (Figure 9) and ErbB4 (Figure 10). The results also revealed the reduced number of communities in the flexible inactive form of ErbB2-WT (Cdk/Src-IF_3_), whereas a larger number of stable interaction networks were observed for the rigid inactive form of ErbB3-WT (Cdk/Src-IF_1_) (Figure 10). To determine characteristic interaction networks that signify different functional states, we mapped the interaction communities in the ErbB kinase structures onto functional dynamics profiles. A critical interaction network conserved in the autoinhibited EGFR conformation was formed by the residues F723-K745-D855-L858 (Figure S5). These interactions ensure the stability of the rigid cluster formed between the αC-helix and a short α-helix of the A-loop, which is a common structural feature of the inactive autoinhibitory form shared by the ErbB kinases. Indeed, a similar interaction community was detected in the inactive ErbB3 (F701-K723-D833-V836) and ErbB4 structures (F704-K726-D836-L839) (Figure S5). These interactions that determine structural stability of the autoinhibitory state involve a contribution of a conserved hydrophobic residue from the A-loop that is targeted by oncogenic mutations in the ErbB kinases (EGFR-L858, ErbB3-V836, and ErbB4-L839). Activating mutations targeting a weak link in the critical interaction network of the autoinhibitory structure may be sufficient to promote destabilization of the inactive state and shift the thermodynamic equilibrium towards the active conformation.

We also observed that that stabilizing communities could be anchored by the R-spine residues from the HRD and DFG motifs. For instance, the local interaction networks in the inactive EGFR included (L828-V774-F856-H835), (V769-M766-F856), (M766-L858-F856), (M825-H835-D837-D896), and (D837-R841-P877) communities (Figure S5). Collectively, these interactions contribute to structural stabilization of the autoinhibitory state by engaging the αC-helix (V765, M766, and V769), the αC-β4-loop (L774), the HRD motif (H835, D837) and DFG motif (D855, F856) in the global interaction network. Of particular interest was the distribution of local communities near the hinge site formed by the αC-helix and the αC-β4-loop that could be involved in coordinating conformational changes during activation. A structurally stable community in this region (V769-M766-F856) is proximal to a weaker interaction cluster formed between the αC-helix residue V769 and αE-helix residues Y827 and R831. The interactions between these residues may represent another “weak” link in the structurally rigid autoinhibited form of EGFR that may be targeted by known activation mutation EGFR-V769L [41].

In the active EGFR form, the emergence of larger communities (F856-L828-V774-V853-I853-M825-H835), (V765-M766-V769-L777-F856), and (V774-Y827-V769-L777) could stabilize the active positions of the αC-helix (V765, M766, and V769) and the αC-β4-loop (V774, L777) (Figure S5). These N-terminal communities are also linked to the C-terminal communities (R841-D837-P877-L858), (R836-L858-V876-D837), (R836-Y869-Y891), and (Y891-M881-W880). Collectively, these stable interaction modules couple the nucleotide and substrate binding sites by linking R836 of the HRD motif with Y869 in the A-loop (primary phosphorylation site), Y891 and W880 from the conserved WMAPE motif in the substrate P+1 loop. Similar interaction networks were observed in the active ErbB4 structure (Figure S5). In this case, interaction communities link the αC-helix (I746, M747, M750), the αC-β4-loop (L755), and the αE-helix (M806, Y808, L809, R812) thus stabilizing the active position of the regulatory αC-helix. The conserved arginine R817 within the HRD motif tethers the A-loop residue L839 (L858 in EGFR) to Y850 (phosphorylation site) via communities (R817-L839-M857-D818) and (R817-Y850-F872). These interaction networks in ErbB4 are highly similar to the respective EGFR communities (R841-D837-P877-L858), (R836-Y869-Y891). A similar reorganization of stabilizing interaction networks during conformational changes is reflective of the conserved activation mechanisms in EGFR and ErbB4 kinases. The community analyses suggested that the R-spine residues could form critical bottlenecks in the interaction networks and correspond to bridging nodes connecting local communities, which may be indicative of their important mediating role in allosteric interaction networks.

### Structure-Based Network Analysis of Allosteric Communications: The Global Centrality of the R-spine Residues

**Figure 11:**
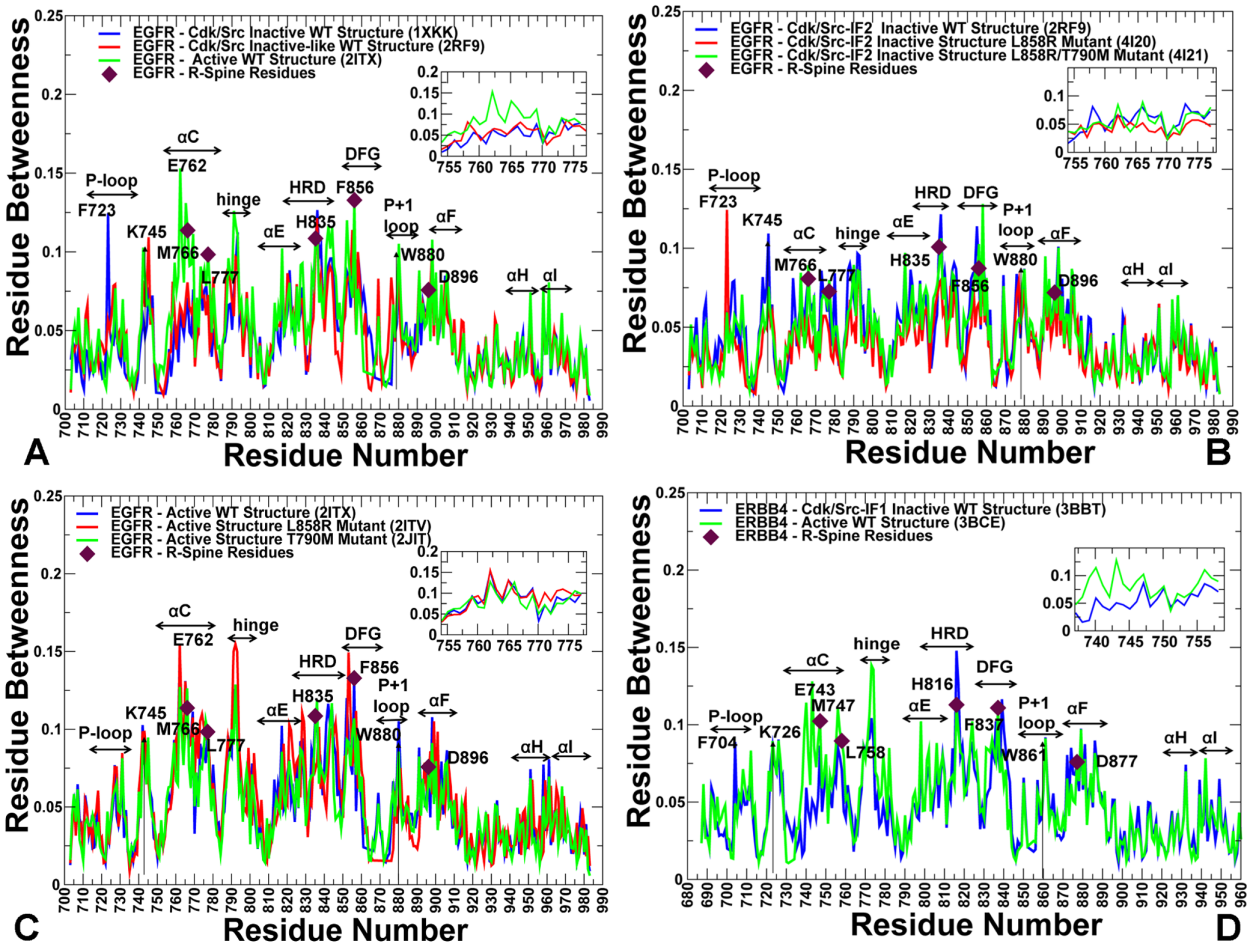
Centrality Analysis of the EGFR and ErbB4 Kinase Domains. (A) The residue-based betweenness profiles of the EGFR-WT structures are shown for Cdk/Src-IF1 (in blue), Cdk/Src-IF2 (in red) and the active conformation (in green). (B) The betweenness profiles of Cdk/Src-IF2 EGFR structures (WT in blue, L858R in red, and L858R/T790M in green). (C) The betweenness profiles of the active EGFR structures (WT in blue, L858R in red, and T790M in green). In (A–C) a close-up view of the EGFR force constant profile in the αC-helix (residues 752–768) and the adjacent αC-β4-loop regions (residues 769–777) is provided as an inset. (D) The betweenness profiles of Cdk/Src-IF1 and active ErbB4 structures are shown in blue and green respectively. A close-up view of the EGFR force constant profile in the αC-helix (residues 752–768) and the adjacent αC-β4-loop regions (residues 769–777) is provided as an inset. The annotated EGFR residues and respective functional regions corresponding to the peaks in the profiles (A–C) included: F723 (P-loop), catalytic pair K745 and E762, M766, L777 (αC-helix), hinge, αE-helix, H835(HRD motif), F856 (DFG motif), W880 (P+1 substrate loop), D896 (αF-helix), αH, and αI helix. The R-spine EGFR residues (M766, L777, H835, F856, D896) are shown by filled maroon-colored diamond symbols. The annotated ErbB4 residues and functional regions in (D) included F704 (P-loop), catalytic pair K726 and E743, M747, L758 (αC-helix), hinge, αE-helix, H816 (HRD motif), F837 (DFG motif), W861 (P+1 substrate loop), D877 (αF-helix), αH, and αI helix. The R-spine ErbB4 residues (M747, L758, H816, F837, and D877) are shown by filled maroon-colored diamond symbols.

We explored the protein structure network analysis to identify global mediating nodes and characterize allosteric communications in the ErbB kinase structures. In this analysis, the average residue centrality (or betweenness index) was computed using the results of MD simulations in different kinase states. The betweenness of a residue node is defined as the number of shortest paths that can go through that node, thus estimating the contribution of the node to the global communication flow in the system. High betweenness nodes can influence the spread of information through the network by facilitating, hindering, or even altering the communication between others. According to our hypothesis, the essential for allosteric signaling residues with high communication capabilities would likely have a significantly higher betweenness as compared to the network average. The ensemble-derived centrality profiles supported this conjecture and revealed key differences between the inactive and active kinase forms. The characteristic feature of the active kinase states is the higher average betweenness, as compared to the inactive structures, and the emergence of sharper peaks corresponding to the R-spine residues (Figure 11). Furthermore, similar peaks in the force constant profiles and centrality distributions of active kinase forms often pointed to the same residues. Some of the characteristic peaks for the inactive EGFR structures corresponded to F723 and L858 residues that are involved in the local community (F723-K745-D855-L858) critical for stability of the autoinhibitory EGFR state. However, these residues are no longer among mediating sites in the active state, as the disintegration of the autoinhibitory lock dissolves the P-loop/A-loop interactions holding the αC-helix in the inactive position.

The characteristic centrality features of the active EGFR structure also included the emergence of strong peaks corresponding to the catalytic residue pair (K745, E762) and ATP-binding site residues. These functional sites are highly central not only compared to the peripheral solvent-exposed residues, but also relative to the core of the catalytic domain. We also detected clear peaks in the EGFR centrality profiles that corresponded to H835 from the HRD catalytic motif, F856 from the DFG motif, W880 from the conserved WMAPE motif in the substrate P+1 loop, and Y869 of the primary phosphorylation site in the A-loop (Figure 11A–C). Similar peaks were seen in the ErbB4 profiles and included respectively functional residues H816, F837, W861, and Y850 (Figure 11D). Notably, the phosporylatable residues Y869 (in EGFR) and Y850 (in ErbB4) are hydrogen-bonded to the mediating HRD-Arg from the catalytic loop, thus stabilizing the active conformation of the A-loop. In the active EGFR and ErbB4 conformations, the high centrality WMAPE motif anchors the substrate binding P+1 loop to the αF-helix, providing a plausible route for communication between allosteric sites. Interestingly, the high centrality residues in the αF-helix included T903 and L907 that contribute to stabilization of the C-spine (V726, A743, L844, V843, V845, L798, T903, and L907) in the EGFR kinase domain. Noteworthy, both the R-spine and C-spine are anchored to the αF-helix, which is a highly stable integrating component of the kinase core. The oncogenic mutations reduced the average residue betweenness in the inactive form (Figure 11B), while this effect was moderately stimulating in the active forms (Figure 11C). The enhanced centrality of the R-spine residues in the oncogenic mutants corroborates with the notion that activating mutations may enhance structural integrity of the hydrophobic spine.

**Figure 12:**
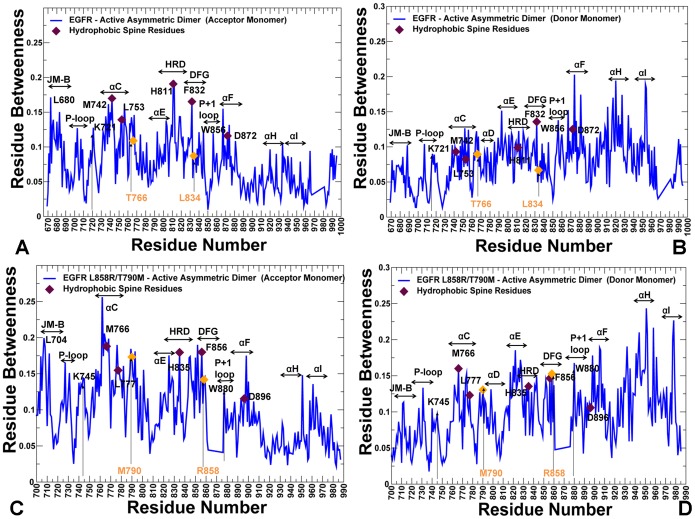
Centrality Analysis of the Active EGFR Dimers. The residue-based betweenness profiles of the active EGFR dimer are shown for EGFR-WT (A, B) and L858R/T790M (C, D). The profiles are shown for the acceptor (left panels A, C) and donor monomers (right panels B, D). The annotated functional regions included JM-B region, P-loop, αC-helix, hinge, αE-helix, HRD motif, DFG motif, substrate binding P+1 loop, αF-helix, αH, and αI helix. The annotated peaks in the profiles reflecting structural stability of the EGFR-WT dimer included L680 (JM-B region), M742, L753, H811, F832, D872 (R-spine residues), and W856 (P+1 substrate loop). The respective peaks in the profile of the EGFR-L858R/T790M dimer corresponded to L704 (JM-B region), M766, L777, H835, F856, and D896 (R-spine residues), and W880 (P+1 substrate loop). The R-spine residues are annotated as maroon-colored diamond symbols and the oncogenic mutation sites are indicated as orange-colored diamond symbols. Note that a different EGFR sequence numbering was adopted in the original crystal structures of the EGFR-WT dimer and L858R/T790M double mutant dimer. We kept the original numbering to avoid confusion in comparisons with the experimental data. In the EGFR-WT structure (A, B); the oncogenic sites correspond to L834 and T766. The R-spine residues in EGFR-WT are M742, L753, H811, F832, and D872. In the crystal structure of the EGFR oncogenic mutant (C, D), the mutated residues correspond to L858R and T790M. The R-spine residues in the mutant structure are M766, L777, H835, F856, and D896.

The important contribution of the R-spine residues becomes even more apparent from the centrality analysis of the regulatory dimers (Figure 12). A strong correspondence between the R-spine residues and the highest peaks of the centrality distribution could be seen in the acceptor monomer of EGFR-WT (Figure 12A). The αC-β4-loop spine residues (M742, L753) along with H811 (HRD motif), F832 (DFG motif), and W856 (WMAPE motif in the P+1 loop) corresponded to the local maxima. The prominent contribution of the JM-B segment was seen in the emerging peak corresponding to L680 in the acceptor monomer (Figure 12A). We observed a number of similar peaks in the donor monomer (Figure 12B), but the high centrality sites shifted to the αH-helix and αI-helix regions that are involved in the extensive intermonomer contacts. The profile of the L858R/T790M dimer revealed interesting peculiarities as we observed considerable changes in mediating capabilities of the mutated residues (Figure 12 C, D). Indeed, the relatively moderate betweenness values for the EGFR-WT residues T766 and L834 (this corresponds to the original residue numbering in the crystal structure) were seen in both acceptor and donor monomers. In the mutant, these sites (T790M and L858R respectively) experienced a significant increase in their centrality level that was especially pronounced in the acceptor monomer (Figure 12C). According to this analysis, mutated sites L858R and T790M may become global mediators of allosteric communications in the oncogenic dimer, and this effect may be especially pronounced in the acceptor monomer. Hence, mutation-induced structural changes in the global interaction network may preferentially enhance allosteric capabilities of the acceptor monomer residues. To summarize, structure-based network analysis revealed a dual role of the R-spine residues as functional hotspots of the kinase activity that may act as key structural stabilizers and regulators of allosteric signaling.

### Allosteric Communication Pathways in the EGFR and ErbB4 Structures: High Centrality Residues Mediate Kinase Signaling

**Figure 13:**
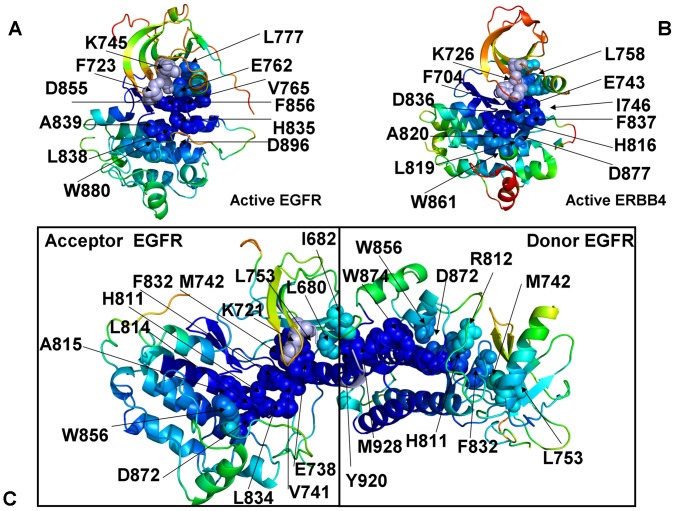
Conformational Allosteric Pathways in the ErbB Kinases. Conformational allosteric pathways between P-loop of the N-terminal lobe and P+1 substrate loop of the C-terminal lobe are shown for the EGFR-WT Kinase domain (Upper Left Panel) and ErbB4 kinase domain (Upper Right Panel). The allosteric pathways are based on the constructed protein structure networks and are determined as the shortest paths between two given residues: F723 in the P-loop and W880 in the P+1 substrate loop for EGFR (Upper left Panel); and between F704 in the P-loop and W861 in the P+1 substrate loop for ErbB4 (Upper Right Panel). The allosteric pathway in the EGFR-WT dimer was obtained as the shortest path in the ensemble of pathways connecting the P-loop of the N-terminal donor monomer with the P+1 substrate loop of the C-terminal acceptor monomer (W856). The residues are colored according to their conformational mobility as in Figures 3–5. The color gradient from blue to red indicates the decreasing structural rigidity (or increasing conformational mobility) of the protein residues. The allosteric pathways are annotated with the contributing residues shown in filled spheres.

Our results have thus far indicated that allosteric signaling between the nucleotide binding site and substrate site may involve the R-spine residues as central mediators of efficient signal communication between the N-terminal and C-terminal lobes. In this section, we analyzed how allosteric signals may be transmitted in the catalytic core. Modeling of communication pathways is directly based on the centrality analysis which generated the ensemble of shortest paths between any pair of residues in the ErbB structures. Our objectives in this analysis were: (a) to map the short communication pathways between high centrality residues in the nucleotide binding site and the P+1 substrate site; (b) to determine the contribution of functional regions (αC-helix, αF-helix, HRD, DFG, P+1 loop) in long-range communication pathways; (c) and to present a mechanistic model of allosteric coupling between the ATP-binding and substrate binding sites. Based on the centrality analysis, we reconstructed shortest pathways connecting the conserved high centrality residues F723 (P-loop) and W880 (P+1 substrate binding site) (Figure 13). These paths connected the P-loop F723 residue via a catalytic pair (K745-E762) with the R-spine residues (M766, L777), subsequently linking V765 (αC-helix), F856 (DFG), H835(HRD), L838, A839, and W880 in the substrate P+1 loop (Figure 13A). We analyzed the topology of communication paths in the context of structural stability and network properties of key residues that mediate these routes. Interestingly, the shortest communication pathways that connect allosteric binding sites in the kinase domain navigated primarily through rigid high centrality nodes. These routes also involved a number of hydrophobic residues (V765, L838, and A839) that may assist central mediating nodes in ensuring the efficiency of allosteric signaling. We also characterized allosteric signaling in the active EGFR dimer by modeling communication pathways that connect the nucleotide binding site in the donor monomer with the substrate site in the acceptor monomer (Figure 13C). Similarly, the optimal routes revealed a geodesic line between the monomers that passed through a set of conserved mediating nodes with the high betweenness value. In the monomer, the allosteric network connected the nucleotide binding site with the R-spine residues and the αE-helix residues (D872, W874). The interactions of these αE-helix residues with the αH-helix (M928, W927, and Y920) in the donor molecule enabled the shortest intermonomer bridge by reaching out to the JM-B residues of the acceptor monomer (L680, I682). The optimal paths then proceeded by linking the JM-B residues and the R-spine residues (M742, L753) of the acceptor monomer, and subsequently connected V741 (αC-helix), F832 (DFG), L834, H811 (HRD), L814, A815, and W856 in the P+1 substrate site of the acceptor (Figure 13C). These results indicated that allosteric communication between distal binding sites may operate via a predominantly single “rigidity propagation path” mechanism [106] in which structurally stable residues act cooperatively to achieve efficient signaling between remote kinase regions in the active state. One could argue that such organization of the interaction networks may also preserve the integrity and efficiency of communication, while achieving a greater resilience against random perturbations. The important conclusion from this analysis is that centrally positioned stable residues that preserve the short paths and ensure the efficiency of allosteric networks in the kinase structures are experimentally known to be important for kinase activity and regulation.

Due to modularity of the interaction networks, residues within the same functional motif may belong to different local communities and perform distinct regulatory roles. The conserved and properly positioned HRD-Asp and DFG-Asp residues are critical for catalytic function and inactivating kinase mutations often target these functional residues. In kinome-wide screens for cancer-causing mutations [107]–[109], some of the ErbB4 mutants, including D818N from the HRD catalytic motif and D836Q of the DFG motif revealed a severely suppressed kinase activity. HRD-His provides critical allosteric connections in the active kinase form by anchoring the R-spine to the central αF-helix, coupling the active site with the catalytic core, and linking the catalytic loop to the A-loop [6]–[12]. Our network analysis indicated that a primary function of the HRD-His may be associated with the assembly of the R-spine and mediating allosteric interactions between the ATP and substrate binding regions, thus making this site indispensable for long-range communication. At the same time, HRD-Arg is primarily involved in bridging local communities in the catalytic loop with the phosphorylated residue in the A-loop and may be less critical for robust signaling between kinase lobes. In this context, we noticed that the HRD-His and DFG-Phe residues from the R-spine are typically surrounded by clusters of stable neighboring residues with an appreciable level of centrality and sufficient communication capacities (Figures 11, 12). This organization of the interaction network may protect critical sites from random perturbations in the fluctuating protein environment. Accordingly, and consistent with the mutagenesis studies [110], [111], our findings suggested that modifications of functional residues which would not interfere with their primary role in allosteric signaling may have a less severe effect on kinase activity, as a dense network of well-connected neighboring residues may preserve the assembled architecture of the R-spine. Indeed, mutations of the kinase residues which undermine structural integrity of the R-spine, could often diminish the kinase activity, and conversely substitutions that strengthen stability of the assembled spine architecture correlated with the enhanced kinase activation [22].

### Conformational Dynamics and Network Analysis of ATP Binding in the EGFR Structures: Allosteric Coupling of ATP and Substrate Sites

We also investigated the effect of ATP binding on conformational dynamics and structural stability of the EGFR structures. We performed MD simulations and a direct comparison of Apo EGFR-WT (pdb id 2GS2) and nucleotide-bound EGFR-WT (pdb id 2ITX). We compared conformational flexibility of the EGFR structures based on the RMSF fluctuations and computed B-factor values (Figure S6). Although the presence of the nucleotide is an important factor contributing to stability of the active kinase, the conformational dynamics of Apo and ATP-bound kinase forms systems was generally quite similar. However, we noticed marginally larger fluctuations distributed across different regions of the ATP-bound EGFR, indicating that the nucleotide binding may cause a subtle redistribution of conformational mobility in the kinase core. Functional dynamics profiles of Apo and ATP-bound EGFR structures (Figure S6) projected onto the essential space of three lowest frequency modes were also similar. However, we noticed minor increases in structural stability of the R-spine residues M766, L777 from the αC-helix in the nucleotide-bound EGFR form. Hence, differences in the conformational dynamics of these systems may be small and rather subtle. While large conformational changes and collective motions can be identified from the normal mode analysis, subtle conformational rearrangements at the side-chain level due to cumulative effect of many residues may be hidden in traditional analyses of MD simulations. Consequently, we proposed that the effects of nucleotide binding on the dynamics and allosteric coupling in the active EGFR structure may be better captured by using a dynamics-based network analysis. In this formulation, the nodes are formed not only by protein residues but also by ligand atoms, so that ATP binding may introduce new edges between residues and partly redistribute the interaction networks.

**Figure 14:**
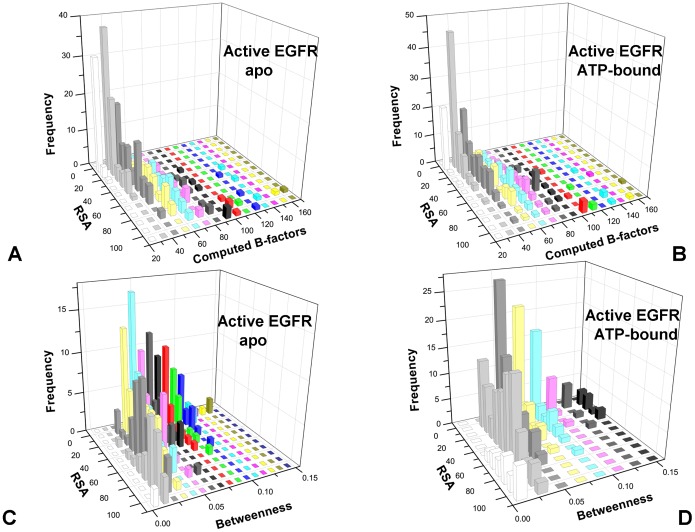
Conformational Mobility and Centrality Profiles: A Comparative Analysis of Apo-EGFR and ATP-bound EGFR Structures. (A, B) Joint distributions of conformational mobility (computed B-factors) and relative solvent accessibility (RSA) are shown for Apo EGFR form (pdb id 2GS2) and ATP-bound active EGFR structure (pdb id 2ITX). Joint distributions of network centrality and RSA parameters are shown for Apo EGFR form (C) and ATP-bound active EGFR structure (D). The MD-based distributions indicated similarity in the conformational mobility and important differences in the network centrality profiles.

**Figure 15:**
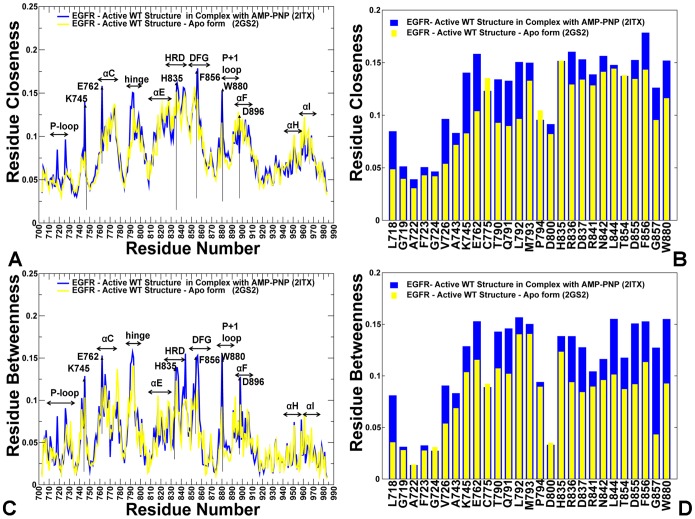
Centrality Analysis of the Apo and ATP-bound EGFR Structures. (A) The residue-based closeness profiles are shown for the Apo form of EGFR-WT (in blue) and ATP-bound EGFR (in green). (B) The residue-based closeness in the Apo-EGGFR (blue bars) and ATP-bound EGFR (green bars) are highlighted for key functional and nucleotide binding site residues, including the catalytic pair (K745, E762), R-spine (H835, F856, and D896), W880 (P+1 substrate loop), P-loop residues (L718,G719,A722,F723), hinge residues (T790,Q791,L792,M793), HRD motif (H835,R836,D837), DFG motif (D855,F856,G857). (C) The residue-based betweenness profiles are shown for the Apo form of EGFR-WT (in blue) and ATP-bound EGFR (in green). (D) The residue-based betweenness in the Apo-EGFR (blue bars) and ATP-bound EGFR (green bars) are highlighted for key functional residues including the R-spine and ATP-binding site residues.

We first constructed joint density distributions of the computed B-factors and RSA values in the Apo and ATP-bound EGFR structures (Figure 14A, B). The distributions appeared to be very similar and reflected a fairly strong correspondence between structural stability and the degree of residue burial. It is quite apparent that this analysis could not detect cumulative allosteric changes that may be induced by ATP binding in the catalytic core. In contrast, we observed important differences between joint distributions computed as a function of residue betweenness and RSA values for Apo-EGFR (Figure 14C) and ATP-bound EGFR (Figure 14D). In these distributions “poor” centrality is typically associated with solvent-exposed residues (high RSA values). We observed that Apo-EGFR is characterized by a dense distribution with a very short “tail” of residues with high betweenness and low RSA values (Figure 14C). On the other hand, in the ATP-bound EGFR structure, we detected a noticeable peak at the end of the distribution tail, revealing that a number of buried and partially exposed residues could attain the increased betweenness values (Figure 14D). According to these observations, ATP-induced modulation of the residue interaction networks may result in the increased centrality of specific residues that are broadly distributed across the kinase core. To elaborate on this point, we compared residue-based closeness and betweenness distributions in the EGFR structures (Figure 15). Both network-based metrics displayed a clear differentiation between Apo-EGFR and ATP-bound EGFR by revealing the increased centrality values and more pronounced distribution peaks in the ATP-bound EGFR structure (Figure 15A, C). Interestingly, these differences were more apparent in the betweenness profile than in the closeness distribution. Accordingly, ATP binding may marginally enhance connectivity of functional residues (closeness), but have a greater effect on allosteric coupling by significantly increasing the centrality of a few major mediating modes (betweenness) and thus improving the efficiency of network communication. A more detailed analysis of the nucleotide-induced differences in residue centrality revealed that the effect may be broadly distributed in the kinase core (Figure 15B, D). The immediate “network-bridging” effect of ATP binding could be seen in the increased centrality of ATP-interacting residues in the P-loop (L718,G719,A722,F723) and hinge residues (T790,Q791,L792,M793) (Figure 15D). The nucleotide binding may also enhance the centrality of key mediating residues H835, R836 (HRD motif), R841, N842, F856 (DFG), and W880 (P+1 loop) that are responsible for allosteric signaling but are not directly connected to the active site. In the preceding section, we showed that most of these centrally positioned residues may be involved in communication pathways between the nucleotide site and the substrate binding site. We also noticed that ATP binding would not necessarily cause a considerable redistribution of mediating sites, but may rather amplify communication capacities of key mediating residues. In this mechanism, by synchronizing “cross-talk” and increasing signaling flow between these mediating sites, ATP binding may improve allosteric coupling of the N-terminal and C-terminal lobes and effectively position the allosteric pocket for substrate binding.

These results are consistent with the NMR studies of PKA kinase [52]–[57], according to which ligand-induced changes are not limited to the active site residues, but may cause chemical shift perturbations in the substrate binding loop. The increased centrality of mediating residues was primarily seen in the hinge region, αC-helix, HRD and DFG motifs, and P+1 substrate loop. The agreement with the experiment is particularly revealing in detecting a strong network-bridging effect of ATP binding on catalytic residues in the binding site (K745, E762) and W880 from the conserved WMAPE motif in the substrate P+1 loop. NMR studies [52]–[57] have shown that the allosteric network and cooperativity of ligand binding in PKA-C can be completely dismantled by a single site mutation (Y204A) of Y204 from the YLAPE motif in the P+1 site. According to our results, the network properties of the corresponding W880 residue in the P+1 loop of EGFR may be allosterically modulated by ATP binding and, therefore, similarly affected by targeted mutations. At the same time, we noticed a counter-effect of ATP binding, manifested in subtle reduction of the residue betweenness (and hence structural stability) across the kinase core (Figure 15A, C). This is consistent with the experimental evidence [52]–[57], suggesting a compensatory increase in mobility of the kinase regions in response to stabilization of the ATP-interacting residues. Hence, small structural changes induced by nucleotide binding may be accompanied by partially increased conformational dynamics in the rigid αE-helix and αF-helix regions, which may be required to activate fast motions in the C-terminal lobe during substrate recognition [56].

In summary, we conclude that conformational dynamics and topology of the interaction networks may be encoded in the ligand-free enzyme. However, the nucleotide binding may induce subtle changes in the interaction networks and enhance allosteric coupling in the active kinase form that is required for catalytic function and substrate binding.

## Discussion

In this section, we discuss the results and implications of our study in the context of a broad range of structural and functional experiments. Conformational dynamics of the multiple functional forms of EGFR demonstrated a marked difference between structural rigidity of the autoinhibited Cdk/Src-IF_1_ structure and flexibility of the alternative Cdk/Src-IF_2_ state. Furthermore, we found that stability of the hydrophobic spine in the autoinhibited, inactive state can be contrasted with the loose and disjointed organization of the R-spine in Cdk/Src-IF_2_ form. These findings were intriguing since the catalytic domains of the oncogenic EGFR mutants adopted an intrinsically mobile Cdk/Src-IF_2_ conformation in the crystal structures [46], [47]. Hence, although conformational landscapes of the EGFR-WT and mutants are topologically similar, the dynamics of conformational changes between the inactive and active states caused by EGFR mutants may be rather specific and unique. These findings indicated that oncogenic mutations may function by modulating the relative populations of the inactive conformations in order to promote kinase activation. We interpreted these results by comparing computational predictions with the recent structure-functional experiments performed for EGFR mutants [46]. In this experimental study, X-ray crystallography and differential scanning calorimetry were used to understand the effect of EGFR mutations on conformational dynamics and thermal stability of various kinase states. The melting temperatures and the enthalpies of denaturation for various kinase forms allowed for a qualitative assessment of protein stability, showing that EGFR-WT is the most stable in its inactive Cdk/Src-IF_1_ form, while the thermal stability of the L858R and L858R/T790M mutants is considerably reduced in their inactive states. The experiments and computation were consistent in demonstrating that EGFR-WT was more structurally stable than the mutated forms of the enzyme as indicated by the higher melting temperature for EGFR-WT [46]. The higher energy required for denaturation is consistent with the more stable conformation of the autoinhibited inactive structure of EGFR-WT. The lower melting temperatures experimentally observed for the L858R and L858R/T790M mutants may reflect a more flexible inactive conformation adopted by EGFR mutants. Our results are consistent with these experiments by asserting that flexibility of oncogenic mutants may compromise the tight interactions seen in the autoinhibited form of EGFR-WT and may reduce the energetic cost of inducing the active conformation. This may provide a mechanism for escaping from the autoinhibitory trap and contribute to uncontrollable kinase activation and the transforming potential of EGFR mutants.

We also found that activating kinase mutations may occur at “soft sites” of the catalytic domain that have an average level of network centrality and are often located at the intersection of high and low stability regions bridging structurally rigid αC-β4-loop and flexible αC-helix. In contrast, inactivating kinase mutations often target catalytically important residues in the HRD and DFG motifs [107]–[109]. In the protein network, these functional residues corresponded to the high centrality sites with the shortest average path length to other protein regions. The optimal communication pathways between the nucleotide and substrate binding sites are also controlled by these nodes and may ensure efficient allosteric signaling in the active kinase state. Hence, residues that are indispensable for kinase activity and signaling may be preferentially centered within the protein structure network. Random mutations may typically compromise local interactions and potentially weaken or even disintegrate a particular community, but would not likely dismantle the integrity of the global interaction network. In contrast, targeted mutations of high centrality mediators could delete the “long-range bridges” that connect distantly located communities, such as allosteric binding sites, leading to fragmentation and potential collapse of the entire network structure. The dependency on these central mediating sites may explain vulnerability of the interaction networks to targeted perturbations of these residues that can abrogate their primary function and consequently lead to a significant loss of kinase activity. Protein structure networks in which highly connected residue nodes with similar networking parameters are interconnected may optimally balance the efficiency of allosteric communications and a greater resilience against random mutations [112]–[114]. We argue that organization of the residue interaction networks in the kinase structures may exhibit elements of modularity that may have evolved to achieve a trade-off between structural stability and the efficiency of allosteric communications.

Our results may also have interesting implications for understanding molecular regulation of protein kinases by the Hsp90 chaperone machinery. The Hsp90 chaperone mediates maturation of many protein kinase clients and supports kinase functional activity that is essential for the integrity of various signaling pathways [115]. The critical determinant that controls functional dependence of client kinases is the preferential Hsp90 binding to the kinases that may be intrinsically unstable in their native folds [116]. Moreover, the strength of the interactions between Hsp90 and kinases strongly correlated with the thermal instability of the kinase domain. Oncogenic kinases can adopt different mechanisms to alleviate negative regulatory processes associated with their intrinsic conformational instability [117]. One of them is the recruitment to the Hsp90 system that protects kinases that are abnormally activated by mutations in cancer cells and would otherwise be prone to aggregation or proteasomal degradation. Unlike EGFR-WT, oncogenic EGFR mutants maintain strong interactions and are dependent on chaperone function for conformational maturation and stability [118]–[120]. Among known ErbB kinases, only ErbB2 could strongly bind to the Hsp90 chaperone in the WT form [121]. In light of our results, oncogenic EGFR variants and ErbB2 are highly dynamic in their inactive states and may readily interconvert with the active form, causing an uncontrollable activity. We argue that oncogenic EGFR mutants may exploit the Hsp90 predisposition for unstable kinase folds to sequester a flexible inactive conformation and promote transformation to the active state. In this mechanism, oncogenic mutants would rely on the Hsp90 dependence for the maintenance of stability and accumulation of the constitutively active form. Consistent with the proposed mechanistic model, Hsp90 function appears to be essential to maintain high-level expression of mutant EGFR in lung cancer cells [118]. Remarkably, ATP-competitive kinase inhibitors may exert their primary effect by antagonizing the Hsp90-kinase interactions and depriving the client kinase of access to the molecular chaperone system [122], [123]. Our results may be relevant to the hypothesis that the Hsp90-kinase interactions may have allowed for diverse kinase functions by protecting the active kinase form and sustaining pressure of detrimental mutations to produce unstable or inactive proteins.

## Materials and Methods

### Structure Preparation

The crystal structure of the ErbB kinases in various conformational states were obtained from the Protein Data Bank (RCSB PDB www.rcsb.org) [124]. A spectrum of simulated crystal structures included the Apo forms, the crystal structures of the kinase-inhibitor complexes, and the crystal structures of the nucleotide-bound complexes. The inactive EGFR crystal structures included the following pdb entries: pdb id 2GS7 (Cdk/Src-IF1 EGFR-WT in complex with AMP-PNP); pdb id 1XKK (Cdk/Src-IF1, EGFR-WT in complex with Lapatinib); pdb id 2RFE (Cdk/Src-IF1, EGFR-WT in complex with a 40-residue MIG peptide); pdb id 2RF9 (Cdk/Src-IF2, EGFR-WT in complex with a 60-residue MIG6 peptide); pdb id 4I20 (Cdk/Src-IF2, Apo EGFR-L858R, V948R); pdb id 4I1Z (Cdk/Src-IF2, Apo EGFR-L858R/T790M, V948R); and pdb id 4I21(Cdk/Src-IF2,EGFR-L858R/T790M in complex with MIG6). The active EGFR crystal structures used in simulations included the following pdb entries: pdb id 2GS2 (active, Apo EGFR-WT); pdb id 2ITX (active, EGFR-WT in complex with AMP-PNP); pdb id 2J6M (active, EGFR-WT in complex with AEE788 inhibitor); pdb id 2ITV (active, EGFR-WT in complex with AMP-PNP); and pdb id 2JIT (active, Apo EGFR-T790M).

The simulated crystal structures of the ErbB kinases also included the following pdb entries: pdb id 3PP0 (Cdk/Src-IF3 Apo ErbB2-WT); pdb id 3KEX (Cdk/Src-IF1, Apo ErbB3-WT), pdb id 3LMG (Cdk/Src-IF1, ErbB3-WT in complex with AMP-PNP); pdb id 3BBW(Cdk/Src-IF1, Apo ErbB4-WT); pdb id 3BBT (Cdk/Src-IF1, ErbB4-WT in complex with Lapatinib); and pdb id 3BCE (active, Apo ErbB4-WT). For simulations of the EGFR regulatory complexes we utilized the crystal structures of an asymmetric, active dimer (pdb id 2GS6) and inactive symmetric dimers (pdb id 2GS6, pdb id 3GT8). ErbB4 kinase adopts an active-like conformation and forms the same asymmetric dimer (pdb id 3BCE) as in the EGFR kinase (pdb id 2GS6). The retrieved structures were examined for missing and disordered segments. The missing residues, unresolved structural segments and disordered loops were modeled with the ModLoop server by preserving the original protein sequence [125], [126].

### MD Simulations and Analysis of Collective Motions

MD simulations of the ErbB kinase crystal structures (each of 50 ns duration) were performed for both monomeric structures of the catalytic domain and the active asymmetric dimers of EGFR-WT, EGFR-L858R/T790M, and ErbB4-WT. We initially conducted simulations of the crystal structures in which crystallographic water molecules, bound inhibitors, nucleotides and cofactors were removed. In addition, we also conducted control simulations of the nucleotide-bound EGFR crystal structures: pdb id 2ITX (active EGFR-WT in complex with AMP-PNP); pdb id 2ITV (active EGFR-WT in complex with AMP-PNP). AMP-PNP was modified into ATP. The employed MD protocol is consistent with the overall setup described in details in our earlier studies [127]. MD simulations were carried out using NAMD 2.6 [128] with the CHARMM27 force field [129], [130] and the explicit TIP3P water model as implemented in NAMD 2.6 [131]. The initial structures were solvated in a water box with the buffering distance of 10 Å. The system was heated from 100 K to 300 K in 30 ps and then cooled down again to 100 K in 30 ps in the NVT ensemble with fixed atom positions except for water and ions. In the following step, the system was heated in the NPT ensemble to 300 K over 30 ps keeping a restraint of 10 Kcal mol-1 Å-2 on protein alpha carbons (C_α_). The system was then equilibrated for 300ps at 300K in the NVT ensemble without restraining forces on the atoms and then for further 300ps at 300K using the NPT ensemble to achieve uniform pressure. An NPT production simulation was run on the equilibrated structures for 50 ns and a time step of 2 fs keeping the temperature at 300 K and constant pressure (1 atm) using Langevin piston coupling algorithm. The van der Waals interactions were treated by using a switching function at 10Å and reaching zero at a distance of 12Å. The SHAKE algorithm [132] was applied to all bonds involving hydrogen atoms. The particle mesh Ewald method [133] was used to treat the long range electrostatic interactions. Principal component analysis of the MD conformational ensembles was based on the extended set of backbone heavy atoms (N, Cα, Cβ, C, O) and used to determine the essential dynamics of the protein systems [134]. The calculations were performed using the CARMA package [135]. The frames are saved every 5 ps, and a total of 10,000 frames were used to compute the correlation matrices for each simulation.

### Force Constant Analysis of Structural Stability

A dynamics-based force constant analysis [96]–[99] is recognized as a robust approach to accurately characterize structurally stable protein regions and describe mechanistic aspects of protein motions at the residue level. The force constant values are obtained from the fluctuations of the mean distance in MD simulations. In this approach, the equilibrium displacements of each residue with respect to the rest of the protein structure are utilized to compute the residue-based force constants effectively measuring the energy cost of the residue fluctuations during simulations. In our model, the force constant for each residue is calculated by averaging the distances between the residues over the MD trajectory using the following expression:
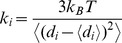
(1)




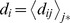
(2)where 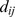 is the instantaneous distance between residue 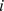 and residue 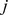, 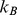 is the Boltzmann constant, 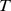 = 300K. 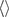 denotes an average taken over the MD simulation trajectory and 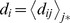 is the average distance from residue 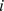 to all other atoms 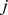 in the protein (the sum over 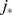 implies the exclusion of the atoms that belong to the residue 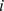). The interactions between the 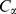 atom of residue 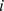 and the 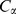 atom of the neighboring residues 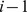 and 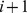 are excluded since the corresponding distances are nearly constant. The inverse of these fluctuations yields an effective force constant that describes the energetic cost of moving an atom with respect to the protein structure.

### Protein Structure Network Modeling: Community Analysis

In the protein structure network analysis, a graph-based representation of proteins was used in which amino acid residues were considered as nodes connected by edges corresponding to the nonbonding residue-residue interactions. The pair of residues with the interaction strength 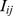 greater than a user-defined cut-off (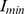) are connected by edges and produce a protein structure network graph for a given interaction strength 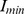. The pair of residues with the interaction strength 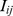 greater than a user-defined cut-off (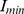) are connected by edges and produce a protein structure network (PSN) graph for a given interaction strength 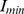. According to the analysis of a large number of protein structures, the optimal interaction strength 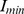 is typically in the range 2–4% [79], [80]. We considered any pair of residues to be connected if 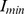 was greater than 3.0%. We adopted a weighted network representation of the protein structure [85]. In this description, both non-covalent connectivity of side chains and residue cross-correlation fluctuation matrix enter as fundamental ingredients in the construction of network graphs.

Protein networks were constructed by incorporating the topology-based residue connectivity and MD-generated maps of residues cross-correlations. According to the adopted model of a protein network, the weight 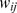 of an edge between nodes 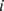 and 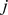 is determined by the dynamic information flow through that edge as measured by the correlation between respective residues. The weight 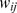 is defined as 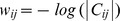 where 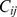 is the element of the covariance matrix measuring the cross-correlation between fluctuations of residues is 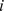 and *j* obtained from MD simulations [85]. The protein structure network analysis of the interaction networks was done using network parameters such as hubs and communities. The hubs are defined as highly connected nodes in the network. If the total number of edges incident on the node (called the degree of a node) is at least four, the node is identified as a local hub. A 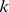-clique is defined as a set of 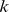 nodes that are represented by the protein residues in which each node is connected to all the other nodes. A 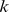-clique community is determined by the Clique Percolation Method [136] as a subgraph containing 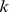-cliques that can be reached from each other through a series of adjacent *k*-cliques.

The construction of protein structure graphs was done with the web-based tool that converts protein structures into graphs (http://vishgraph.mbu.iisc.ernet.in/GraProStr/). Computation of the network parameters was performed using the Clique Percolation Method as implemented in the CFinder program [137]. The residue interaction communities were considered to be dynamically stable if these networks remained to be intact in more than 75% of the ensemble conformations. We also evaluated the propensity of residues from the interaction communities to function as stabilization centers. Stabilizing residues in protein structures were identified using a combination of hydrophobicity, long-range order, stabilization center index and conservation score as described in [138]. The computations were performed using web-based servers SRide and Scide [139].

### Centrality Analysis

Using the constructed protein structure networks, we computed the global centrality measure such as residue-based betweenness. This parameter is based on the determination of the shortest paths between two given residues. Betweenness quantifies the number of times a node acts as a bridge along the shortest path between two other nodes. The betweenness measures the frequency of a given residue to belong to all shortest path pairs within the protein structure. The length of a path 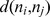 between distant nodes 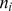 and 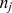 is the sum of the edge weights between the consecutive nodes 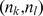 along the path:
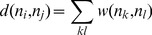
(3)


The shortest paths between two residues are determined using the Floyd–Warshall algorithm [140] that compares all possible paths through the graph between each pair of residue nodes. At the first step, the distance between connected residues was considered to be one, and the shortest path was identified as the path in which the two distant residues were connected by the smallest number of intermediate residues. Network graph calculations were performed using the python module Network [141]. To select the shortest paths that consist of dynamically correlated intermediate residues, we considered the short paths that included sufficiently correlated (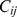 = 0.5–1.0) intermediate residues. This procedure was adopted from previous studies [72], [73] which defined an ensemble of suboptimal pathways connecting spatially separated sites based on the tolerance threshold for the edge weight of connecting residues 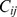 = 0.5. The degree of a node is a centrality measure of the local connectivity in the interaction network. The degree of residue 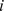 is the number of its direct connections to other residues and is computed as follows:
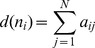
(4)

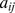
 is the element of adjacency matrix 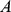; 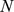 is the total number of nodes in the residue interaction network.

The closeness of residue 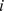 is defined as the inverse of the average shortest path (geodesic distance) from residue 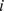 to all other residues in the network. Residues with shorter geodesic distances to the remaining residues typically have higher closeness values. The normalized closeness values can be calculated as follows:
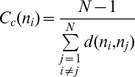
(5)Here, 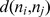 is the shortest path from node 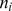 to node 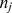. 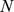 is the total number of nodes.

The betweenness of residue 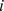 is defined to be the sum of the fraction of shortest paths between all pairs of residues that pass through residue *i*:
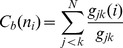
(6)where 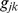 denotes the number of shortest geodesics paths connecting 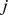 and *k,* and 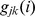 is the number of shortest paths between residues 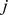 and *k* passing through the node 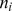. Residues with high occurrence in the shortest paths connecting all residue pairs have a higher betweenness values. The normalized betweenness of residue 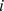 can be expressed as follows:
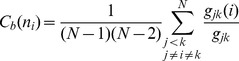
(7)

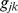
 is the number of shortest paths between residues 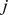 and k; 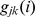 is the fraction of these shortest paths that pass through residue 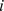.

## Supporting Information

Figure S1 **Atom-Based Equilibrium Fluctuations of the ErbB Kinases.** The computed B-factors describe time-averaged fluctuations of heavy atoms obtained from simulations of (A) Cdk/Src-IF1 EGFR-WT (pdb id 1XKK, in blue), (B) Cdk/Src-IF2 EGFR-WT (pdb id 2RF9, in red), (C) active EGFR-WT (pdb id 2ITX, in green), (D) active EGFR-L858R (pdb id 2ITV, in red), (E) active EGFR-T790M (pdb id 2JIT, in green), (F) inactive EGFR-L858R (pdb id 4I20, in red), (G) inactive EGFR-L858R/T790M (pdb id 4I21, in green), (H) inactive ErbB4-WT (pdb id 3BBT, in green), (I) active ErbB4-WT (pdb id 3BCE, in maroon). The colors correspond to the respective residue-based fluctuation plots in Figure 2.
(TIF)

Figure S2 **Scatter Graphs of the Force Constant and Relative Solvent Accessibility Parameters.** The scatter graphs between the force constant (a dynamics-based residue connectivity measure) and RSA values (an energetics-based estimate of residue solvent accessibility) values are shown for Cdk/Src-IF1 EGFR-WT (A), active EGFR-WT (B), Cdk/Src-IF2 EGFR-WT (C) and Cdk/Src-IF2 EGFR-L858R/T790M (D). The positions of the R-spine residues are indicated by filled squares colored in red. The correlation coefficient values are also shown. The stability of the R-spine residues in the Cdk/Src-IF1 and active EGFR forms can be distinguished from other residues as they are characterized by low RSA values (RSA<10%), and high force constants values.
(TIF)

Figure S3 **The Residue-Based Local Contact Density in the EGFR and ErbB4 Kinase Domains.** The local contact density derived from structure-based network analysis is shown for the Cdk/Src-IF1 and active states of EGFR (A, B) and ErbB4 (C, D). The profiles highlight the local density for well-connected residues with a number of directly interacting residues) exceeding the threshold of four. The residue nodes corresponding to the R-spine residues are indicated by green-colored filled diamond symbols. The R-spine residues in EGFR are M766, L777, H835, F856, and D896. In the ErbB4 structures, the spine residues are M747, L758, H816, F837, and D877. Note that the R-spine residues have a high local connectivity in both inactive and active states.
(TIF)

Figure S4 **The Residue-Based Local Contact Density in the EGFR and ErbB4 Active Dimers.** The local contact density derived from structure-based network analysis is shown for EGFR-WT (A, B) and L858R/T790M (C, D). The profiles are shown for the acceptor (left panels A, C) and donor monomers (right panels B, D). The R-spine residues are indicated by green-colored filled diamonds. The interfacial residues are shown in red-colored filled circles. Note that the inter-monomer high connectivity residues in the acceptor monomer are assembled in the αC-helix and JM-B regions. In the donor monomer the highly connected residues belong mostly to the interfacial αH-helix.
(TIF)

Figure S5 **Structural Mapping of the Interaction Networks in the EGFR and ErbB4 Kinases.** Structural maps of the interaction communities in the inactive Cdk/Src-IF1 form of EGFR (A), active EGFR form (B), the inactive Cdk/Src-IF1 form of ErbB4 (C), and active ErbB4 form (D). The protein residues that form local communities are shown in spheres. The interaction communities that are characteristic of the inactive and states are annotated and depicted by circles. The color gradient from blue to red indicates the decreasing structural rigidity (or increasing conformational mobility) of protein residues.
(TIF)

Figure S6 **Conformational Dynamics of the Apo and ATP-bound EGFR Structures.** (A) The computed B-factors describe time-averaged residue fluctuations obtained from simulations of Apo EGFR-WT (pdb id 2GS2, in red) and nucleotide-bound EGFR-WT (pdb id 2ITX, in blue). Conformational mobility profiles of Apo-EGFR (B) and ATP-bound EGFR (C) projected on the essential space of the three lowest frequency modes. The backbone heavy atoms (N,C_α_,C_β_,C,O) were employed for the PCA computations. The color gradient from blue to red indicates the decreasing structural rigidity (or increasing conformational mobility) of the protein residues and refers to an average value over the backbone atoms in each residue. The functional kinase regions αC-helix, αC-β4-loop, and αE-helix as well as the R-spine residues are annotated and their positions are indicated by arrows. Conformational mobility profiles were obtained from simulations of complete structures, where unresolved segments and disordered loops were modeled with the ModLoop server [127],[128]. These profiles were mapped onto the original crystal structures of EGFR for clarity of presentation as in Figures 3, 4.
(TIF)
