# Supplementary figures and images for: Structure-Based Network Analysis of Activation Mechanisms in the ErbB Family of Receptor Tyrosine Kinases: The Regulatory Spine Residues Are Global Mediators of Structural Stability and Allosteric Interactions
Source: PLoS One. 2014 Nov 26;9(11):e113488. doi: 10.1371/journal.pone.0113488 (PMC4245119; doi:10.1371/journal.pone.0113488)

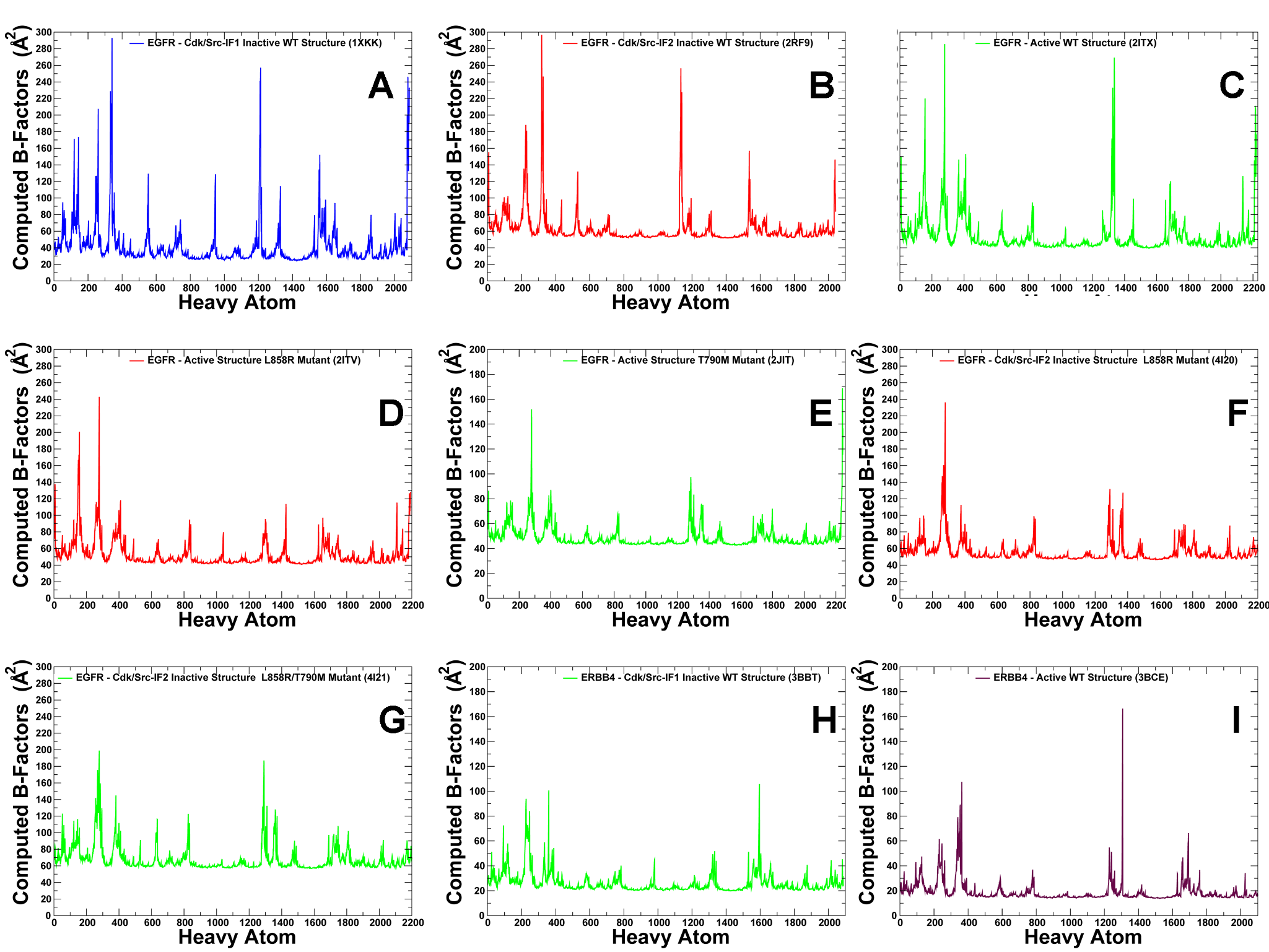

Supplement: Figure S1 — Atom-Based Equilibrium Fluctuations of the ErbB Kinases. The computed B-factors describe time-averaged fluctuations of heavy atoms obtained from simulations of (A) Cdk/Src-IF1 EGFR-WT (pdb id 1XKK, in blue), (B) Cdk/Src-IF2 EGFR-WT (pdb id 2RF9, in red), (C) active EGFR-WT (pdb id 2ITX, in green), (D) active EGFR-L858R (pdb id 2ITV, in red), (E) active EGFR-T790M (pdb id 2JIT, in green), (F) inactive EGFR-L858R (pdb id 4I20, in red), (G) inactive EGFR-L858R/T790M (pdb id 4I21, in green), (H) inactive ErbB4-WT (pdb id 3BBT, in green), (I) active ErbB4-WT (pdb id 3BCE, in maroon). The colors correspond to the respective residue-based fluctuation plots in Figure 2. (TIF) [file pone.0113488.s001.tif]

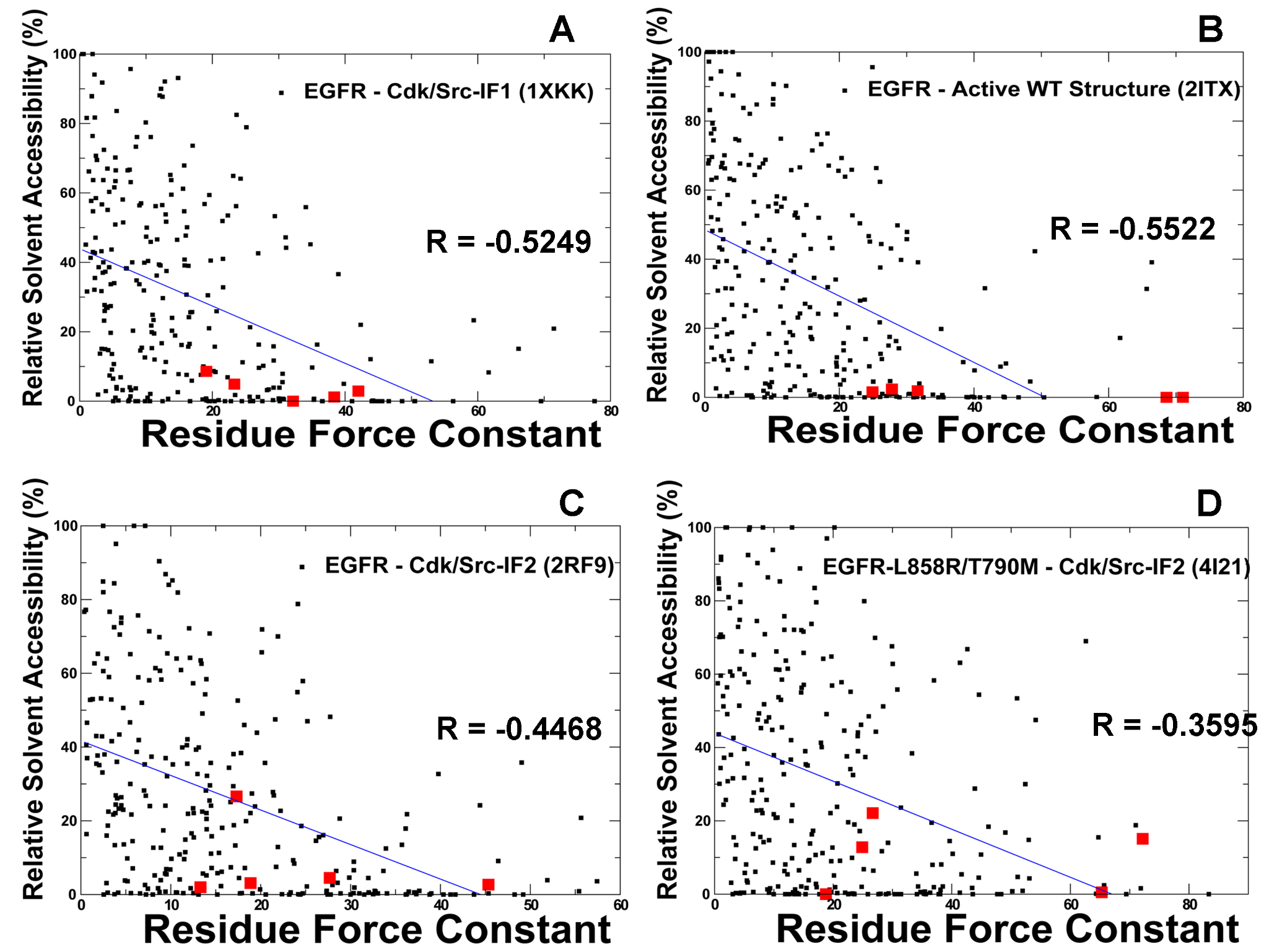

Supplement: Figure S2 — Scatter Graphs of the Force Constant and Relative Solvent Accessibility Parameters. The scatter graphs between the force constant (a dynamics-based residue connectivity measure) and RSA values (an energetics-based estimate of residue solvent accessibility) values are shown for Cdk/Src-IF1 EGFR-WT (A), active EGFR-WT (B), Cdk/Src-IF2 EGFR-WT (C) and Cdk/Src-IF2 EGFR-L858R/T790M (D). The positions of the R-spine residues are indicated by filled squares colored in red. The correlation coefficient values are also shown. The stability of the R-spine residues in the Cdk/Src-IF1 and active EGFR forms can be distinguished from other residues as they are characterized by low RSA values (RSA<10%), and high force constants values. (TIF) [file pone.0113488.s002.tif]

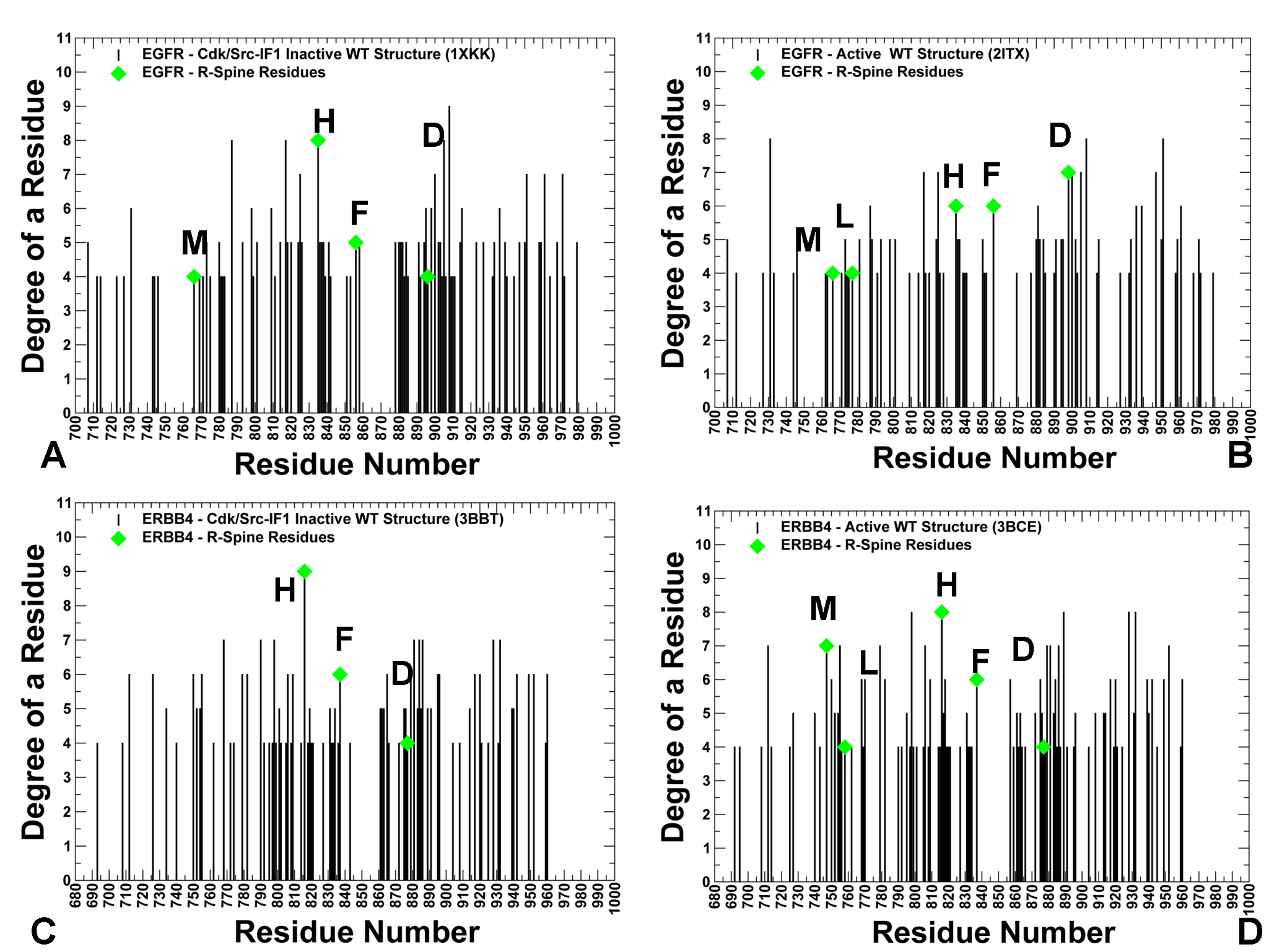

Supplement: Figure S3 — The Residue-Based Local Contact Density in the EGFR and ErbB4 Kinase Domains. The local contact density derived from structure-based network analysis is shown for the Cdk/Src-IF1 and active states of EGFR (A, B) and ErbB4 (C, D). The profiles highlight the local density for well-connected residues with a number of directly interacting residues) exceeding the threshold of four. The residue nodes corresponding to the R-spine residues are indicated by green-colored filled diamond symbols. The R-spine residues in EGFR are M766, L777, H835, F856, and D896. In the ErbB4 structures, the spine residues are M747, L758, H816, F837, and D877. Note that the R-spine residues have a high local connectivity in both inactive and active states. (TIF) [file pone.0113488.s003.tif]

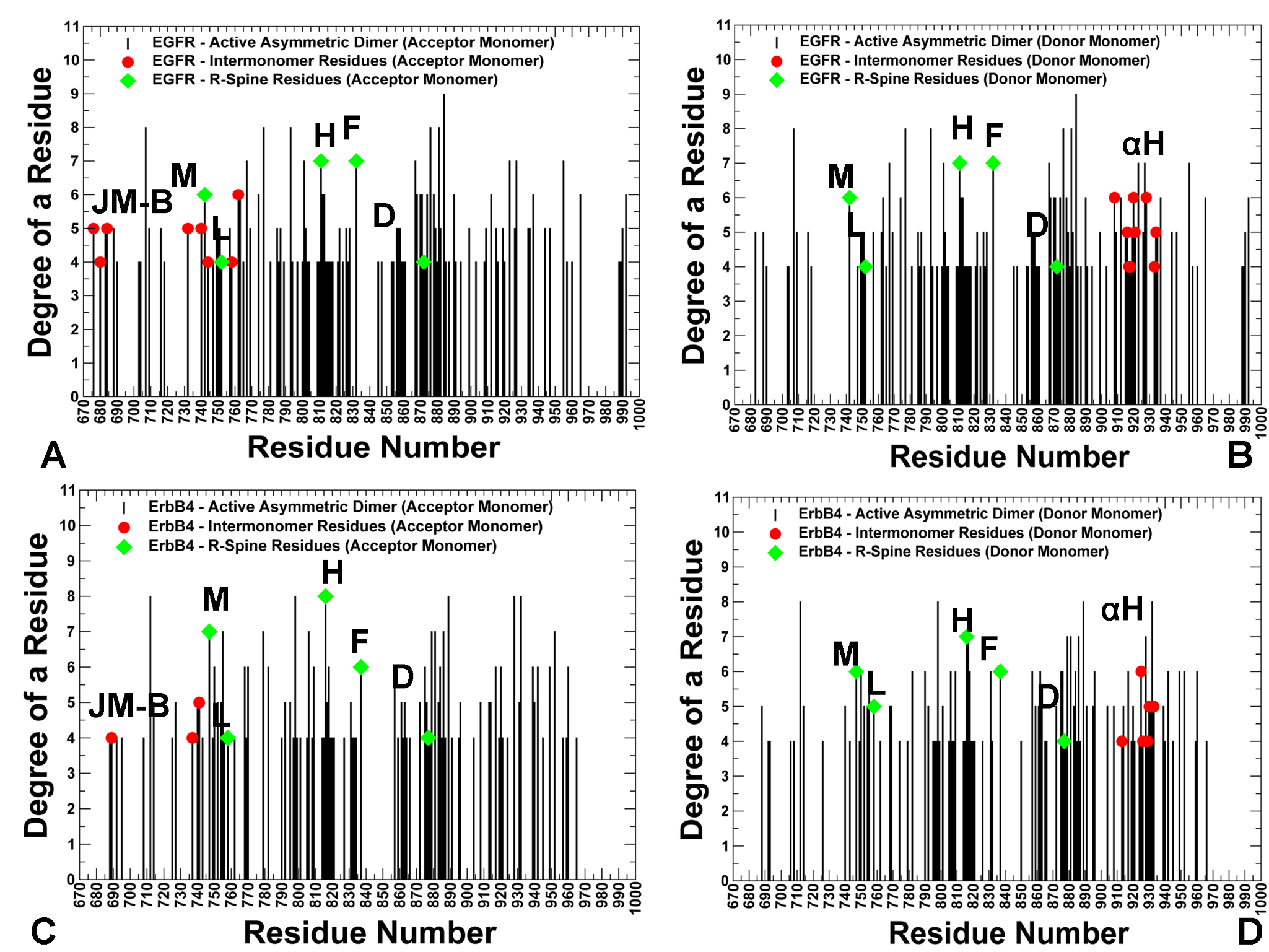

Supplement: Figure S4 — The Residue-Based Local Contact Density in the EGFR and ErbB4 Active Dimers. The local contact density derived from structure-based network analysis is shown for EGFR-WT (A, B) and L858R/T790M (C, D). The profiles are shown for the acceptor (left panels A, C) and donor monomers (right panels B, D). The R-spine residues are indicated by green-colored filled diamonds. The interfacial residues are shown in red-colored filled circles. Note that the inter-monomer high connectivity residues in the acceptor monomer are assembled in the αC-helix and JM-B regions. In the donor monomer the highly connected residues belong mostly to the interfacial αH-helix. (TIF) [file pone.0113488.s004.tif]

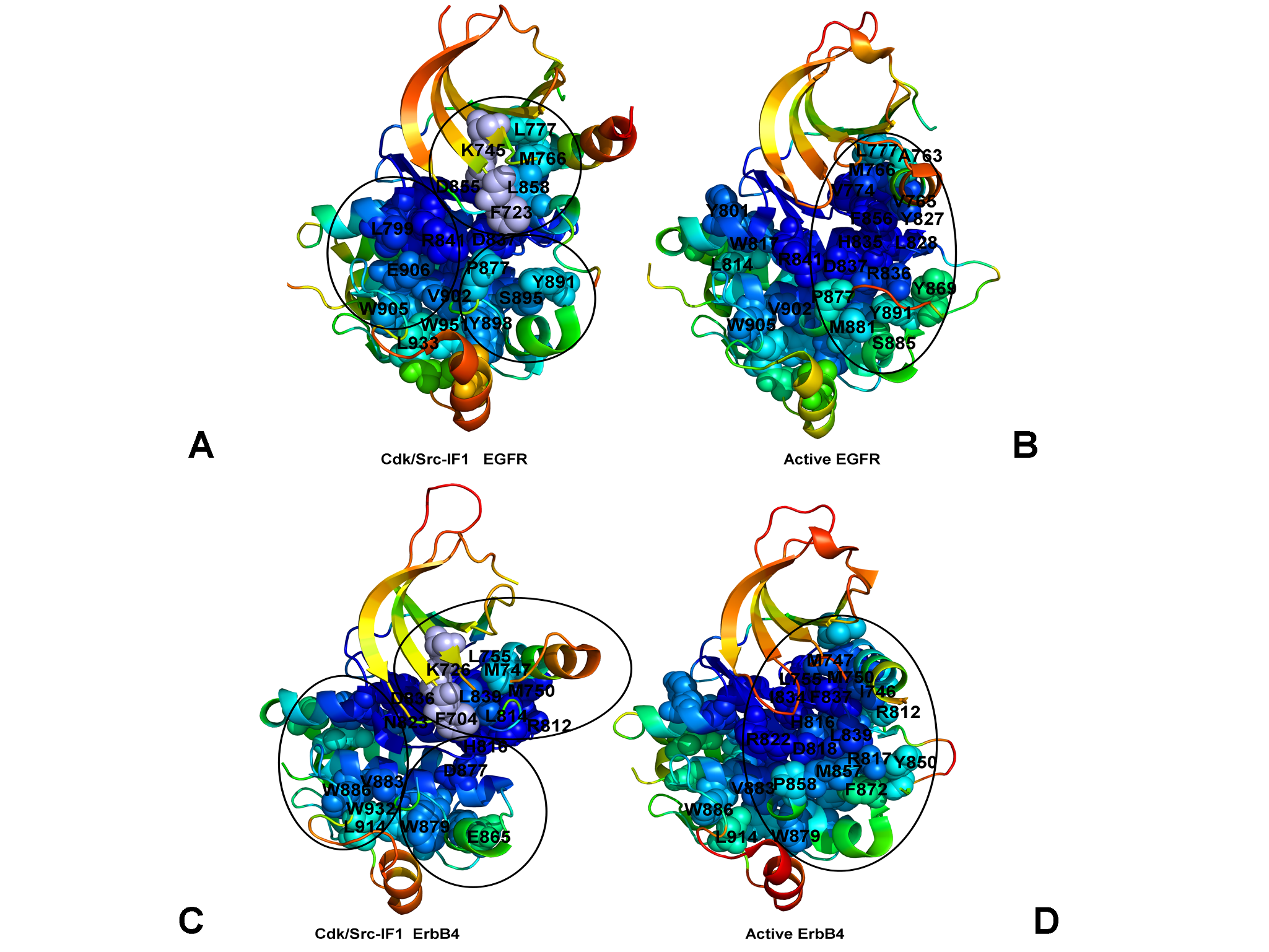

Supplement: Figure S5 — Structural Mapping of the Interaction Networks in the EGFR and ErbB4 Kinases. Structural maps of the interaction communities in the inactive Cdk/Src-IF1 form of EGFR (A), active EGFR form (B), the inactive Cdk/Src-IF1 form of ErbB4 (C), and active ErbB4 form (D). The protein residues that form local communities are shown in spheres. The interaction communities that are characteristic of the inactive and states are annotated and depicted by circles. The color gradient from blue to red indicates the decreasing structural rigidity (or increasing conformational mobility) of protein residues. (TIF) [file pone.0113488.s005.tif]

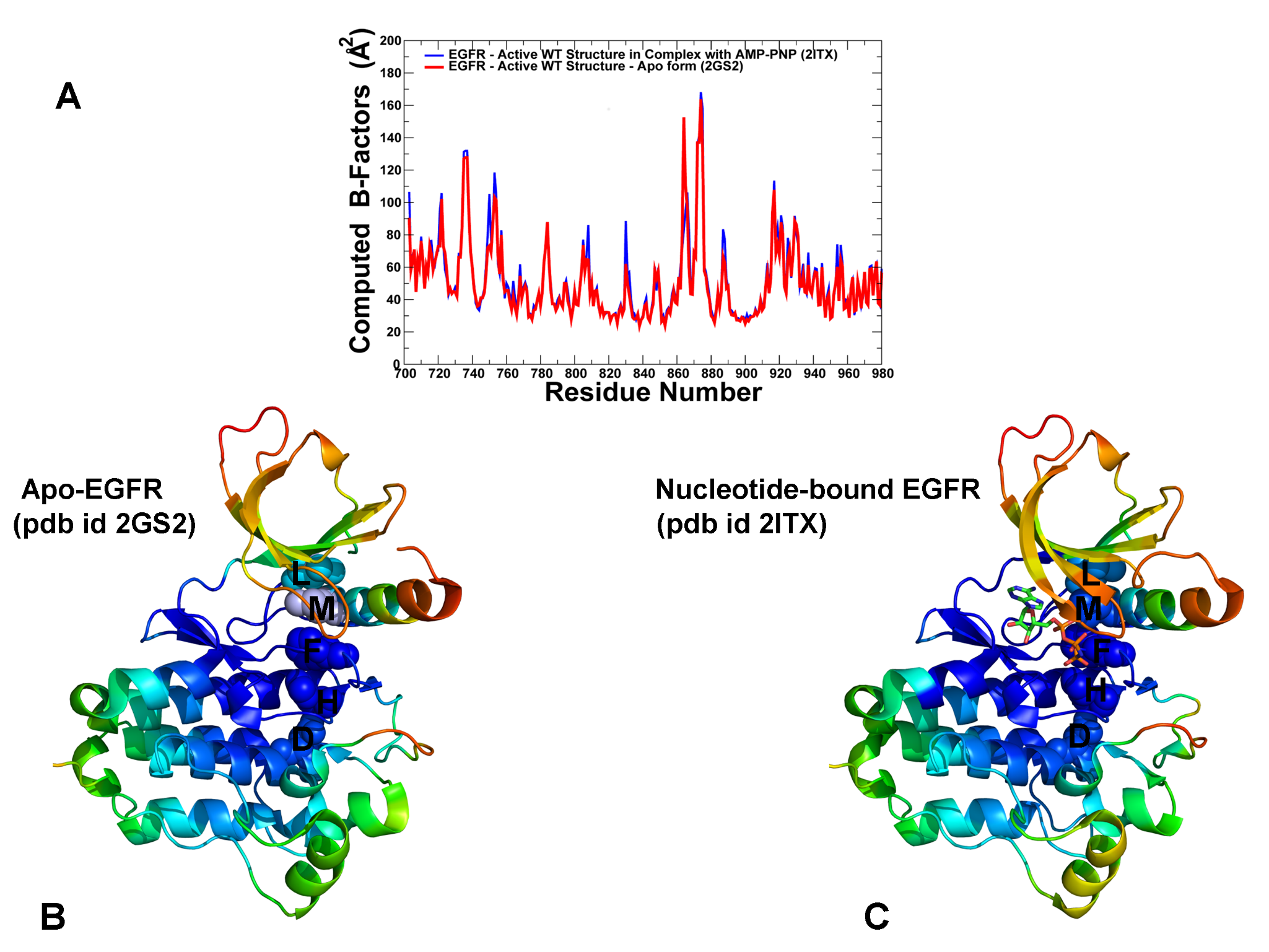

Supplement: Figure S6 — Conformational Dynamics of the Apo and ATP-bound EGFR Structures. (A) The computed B-factors describe time-averaged residue fluctuations obtained from simulations of Apo EGFR-WT (pdb id 2GS2, in red) and nucleotide-bound EGFR-WT (pdb id 2ITX, in blue). Conformational mobility profiles of Apo-EGFR (B) and ATP-bound EGFR (C) projected on the essential space of the three lowest frequency modes. The backbone heavy atoms (N,Cα,Cβ,C,O) were employed for the PCA computations. The color gradient from blue to red indicates the decreasing structural rigidity (or increasing conformational mobility) of the protein residues and refers to an average value over the backbone atoms in each residue. The functional kinase regions αC-helix, αC-β4-loop, and αE-helix as well as the R-spine residues are annotated and their positions are indicated by arrows. Conformational mobility profiles were obtained from simulations of complete structures, where unresolved segments and disordered loops were modeled with the ModLoop server [127],[128]. These profiles were mapped onto the original crystal structures of EGFR for clarity of presentation as in Figures 3, 4. (TIF) [file pone.0113488.s006.tif]
